# Unsupervised method for representation transfer from one brain to another

**DOI:** 10.3389/fninf.2024.1470845

**Published:** 2024-11-28

**Authors:** Daiki Nakamura, Shizuo Kaji, Ryota Kanai, Ryusuke Hayashi

**Affiliations:** ^1^Human Informatics and Interaction Research Institute, National Institute of Advanced Industrial Science and Technology, Ibaraki, Japan; ^2^Institute of Mathematics for Industry, Kyushu University, Fukuoka, Japan; ^3^Araya Inc., Tokyo, Japan

**Keywords:** representation transfer, artificial neural networks, fMRI, image reconstruction, brain-to-brain communication, brain-machine interface, brain-computer interface

## Abstract

Although the anatomical arrangement of brain regions and the functional structures within them are similar across individuals, the representation of neural information, such as recorded brain activity, varies among individuals owing to various factors. Therefore, appropriate conversion and translation of brain information is essential when decoding neural information using a model trained using another person’s data or to achieving brain-to-brain communication. We propose a brain representation transfer method that involves transforming a data representation obtained from one person’s brain into that obtained from another person’s brain, without relying on corresponding label information between the transferred datasets. We defined the requirements to enable such brain representation transfer and developed an algorithm that distills the assumption of common similarity structure across the brain datasets into a rotational and reflectional transformation across low-dimensional hyperspheres using encoders for non-linear dimensional reduction. We first validated our proposed method using data from artificial neural networks as substitute neural activity and examining various experimental factors. We then evaluated the applicability of our method to real brain activity using functional magnetic resonance imaging response data acquired from human participants. The results of these validation experiments showed that our method successfully performed representation transfer and achieved transformations in some cases that were similar to those obtained when using corresponding label information. Additionally, we reconstructed images from individuals’ data without training personalized decoders by performing brain representation transfer. The results suggest that our unsupervised transfer method is useful for the reapplication of existing models personalized to specific participants and datasets to decode brain information from other individuals. Our findings also serve as a proof of concept for the methodology, enabling the exchange of the latent properties of neural information representing individuals’ sensations.

## 1 Introduction

Acquiring information from the brain not only contributes to understanding the neurological mechanisms underlying our perceptions and cognitive processes but also has the potential to enable smoother and more enriched communication by directly transmitting sensations and intentions. Although, humans typically communicate with others using language and non-verbal cues, such as facial expressions or gestures, limitations arise when attempting to express sensations or convey concepts that are beyond the scope of these modalities. Using neural information that represents our sensations and ideas offers the possibility of transcending the limitations of conventional communication.

Various studies have used neural information representing sensations toward practical applications (e.g., the brain-machine interface), alongside basic science, to understand the brain itself. These include the reconstruction of observed artificial experimental stimuli (Kamitani and Tong, 2005; Miyawaki et al., 2008), natural images (Wen et al., 2018; Shen et al., 2019a; Shen et al., 2019b; Ozcelik and VanRullen, 2023), and natural movies (Nishimoto et al., 2011) using functional magnetic resonance imaging (fMRI), and the extraction of object categories of observed images from intracranial local field potentials in patients with epilepsy (Liu et al., 2009). Additionally, we have successfully reconstructed images from neural activity recorded from the monkey brain (Hayashi and Kawata, 2018). Furthermore, methods have been proposed to directly link one brain to another for the purpose of communication (Pais-Vieira et al., 2013). Recent human studies using a non-invasive approach have combined brain activity recordings using electroencephalography (EEG) with neural stimulation techniques using transcranial magnetic stimulation and demonstrated that the decision of one individual based on binary choices can be transmitted to another individual (Grau et al., 2014; Jiang et al., 2019). Lee et al. (2017) demonstrated brain-to-brain transmission of sensorimotor information in humans using EEG signals and focused ultrasound stimulation.

When using neural information for communication, it is crucial that the information is appropriately converted and translated between participants to account for the inter-individual variation in the representation of neural information. For example, although the anatomical arrangement of brain areas is similar across individuals, the exact location and size of specific structures or regions vary among individuals. Similarly, although functional structures within a brain area are comparable across individuals, such as retinotopy in the visual cortex (Fishman, 1997; Wandell et al., 2005), somatotopy in the motor and somatosensory cortices (Penfield and Boldrey, 1937), and tonotopy in the auditory cortex (Romani et al., 1982), the arrangement of individual neurons responsible for a particular function is not identical across participants. Furthermore, differences in recording devices, recorded sensory modalities, and various other conditions/situations among individuals must be accounted for when applying transformations based on transmitting information.

If paired brain data in response to identical stimuli are available for two participants, determining a transformation function between two datasets is relatively straightforward using machine learning methods, such as canonical correlation analysis, which extracts the covariation components of paired samples (Hotelling, 1936; Michalke et al., 2023). In addition, “hyper-alignment” is widely used to obtain common representational patterns across participants from paired fMRI data (Haxby et al., 2020). Modality conversion has also been performed previously between fMRI data and EEG data using the correspondence of the two data (Cheng et al., 2021).

However, corresponding label information related to recorded brain activity may not always be available when transforming brain representations among numerous participants. If the stimulus dataset is not standardized, brain activity may be measured using different stimulus sets, which risks a lack of exact correspondence of the presented stimuli at the individual-stimuli or category level among participants. However, these stimuli may still cover some common topics, contents, and concepts, potentially serving as a coarse-level correspondence that cannot be explicitly defined. It is also considered that the standard stimulus dataset for calibrating the transfer function may change over time. If brain representation transfer can be conducted without the use of label information, it will be possible to adapt to changes in the stimulus dataset in a flexible manner and effectively utilize previously measured data (e.g., by conducting recalibration without remeasurement).

In this paper, we propose an unsupervised method for brain representation transfer that can be performed even when there is no corresponding label information across participants in advance. This unsupervised transfer method was applied under the assumption that the similarities and dissimilarities of relationships in the latent properties of the brain data have certain commonalities across participants. To support this assumption, it is known that neurons within specific regions of the visual cortex selectively respond to certain image features depending on the brain area, such as orientation selectivity (Hubel and Wiesel, 1962), object selectivity (Haxby et al., 2001; Carlson et al., 2003; Kriegeskorte et al., 2008b), color selectivity (Komatsu et al., 1992), and texture selectivity (Okazawa et al., 2015). Therefore, the relationships between neural responses obtained from these neural populations are expected to reflect the similar and dissimilar relationships among their selective image features, regardless of the participant. In our proposed method, we applied machine learning techniques related to dimensionality reduction and unsupervised object matching, both of which rely on similarity relationships among data. We then established transformations of inter-individual neural information representation based on the object-matching result. To validate our proposed method, we conducted two experiments: one experiment used data obtained from an artificial neural network (ANN) as a visual cortex model, and the other experiment used data derived from actual human brains.

## 2 Materials and methods

The main purpose of this study was to propose an algorithm for brain representation transfer without corresponding label information, with the aim of effectively transmitting information represented in the brain between participants. Our basic assumption for achieving this transmission is that the similar and dissimilar relationships in the latent properties of the data are common among participants.

In this section, we first outline the requirements for the brain representation transfer algorithm and introduce the implemented algorithms. We then describe the conditions of two validation experiments for the proposed algorithms, including the datasets, model parameters, and evaluation methods.

### 2.1 Algorithm requirements for brain representation transfer

We identified three requirements that the unsupervised brain representation transfer algorithm should satisfy, especially for the purpose of transmitting individual sensations. Firstly, neural activity data recorded by electrophysiological and/or neuroimaging techniques contain individual differences or modality-specific features that are irrelevant to the transfer. Additionally, the data are affected by measurement noise, motion artifacts, and inherent fluctuations in brain activity. These issues can typically be overcome through dimensionality reduction because neuronal data include redundant information across dimensions in terms of the common latent property we aim to extract. Therefore, we defined requirement (i) the algorithm for brain representation transfer should include a robust dimensionality reduction function that preserves similarity and dissimilarity structures while remaining effective against various types of noise and individual differences. Secondly, in cases where there is no direct correspondence between individual samples in two datasets, the concept of “cycle consistency” becomes crucial in translation tasks. This concept ensures that the translation process between the domains of two datasets is reversible when performed in both directions. In the field of computer vision, this constraint is considered effective for learning a transfer function in an image-to-image transfer task (Zhu et al., 2017); the learned mapping becomes more robust and ensures that the generated outputs are coherent and relevant to the original inputs. This concept is also key constraint in brain representation transfer tasks, where the transformation process is considered bidirectional for communication purposes. Therefore, we defined requirement (ii) the algorithm for brain representation transfer comprises a reversible transformation that satisfies cycle consistency. Thirdly, we envisioned that our proposed method would be used for decoding transmitted neural information and communication. Thus, we defined requirement (iii) the brain representation transfer algorithm should be compatible with machine learning techniques that have been applied to the decoding of neural information. Specifically, we assumed that the transfer algorithm could be implemented as a multi-layered neural network, which is known to be an effective model for information processing in the visual cortex and is widely used for generating images from neural activity data.

We developed the brain representation transfer method by combining several algorithms that satisfy the above three requirements: an algorithm for embedding individual neural information using personalized encoders that preserve the similarity structure of the latent property at the individual level, and an algorithm for transforming neural information between participants by utilizing the commonalities in the similarity structure of the latent properties across participants.

We initially considered using instance learning (Wu et al., 2018) as a learning rule for the algorithm that satisfies requirements (i) and (iii). Instance learning is a variant of contrastive learning (Oord et al., 2018; Chen et al., 2020; Wang and Isola, 2020) that preserves similarity and dissimilarity structures. Contrastive learning is a machine learning technique that embeds similar data (i.e., positive samples) near each other and dissimilar data (i.e., negative samples) far apart in latent space (a hypersphere is typically used as the embedding space). In instance learning, positive samples are identical data obtained through data augmentation, whereas negative samples are other training data excluding the positive samples. Therefore, it is possible to perform dimensionality reduction while preserving the similarity and dissimilarity structures by embedding data into a lower-dimensional space through instance learning. Unlike other unsupervised algorithms, such as t-distributed stochastic neighbor embedding (t-SNE; Maaten and Hinton, 2008) and multi-dimensional scaling (Kruskal, 1964a,1964b), the instance learning framework can flexibly embed novel data that have not been used during the training phase. By exploiting these advantages, we demonstrate the robustness of instance learning against noise in the experiments in Section “3.1 Noise robustness through data augmentation during instance learning.”

Next, we focused on the rotational and reflection (i.e., mirror-flip) transformation, which is a reversible transformation, to satisfy requirement (ii) of the transfer algorithm. If the data satisfy our assumption, it is expected that the latent variables embedded by instance learning will also exhibit common similar and dissimilar relationships across participants. In addition, because we embedded the data into a hypersphere using instance learning, we can expect the arrangement of the embedded data to exhibit rotational and reflective symmetry between participants. Therefore, we used a rotation and reflection matrix (i.e. an orthogonal matrix) as the transformation for embedded data, among various reversible transformations, such as both linear and non-linear transformations. However, determining the orthogonal matrix requires correspondence of embedded data between participants, and we assumed the scenario in which such correspondence information is not explicitly available. To address this problem, we adopted kernelized sorting (Quadrianto et al., 2010), which is an unsupervised object-matching algorithm based on the similarity-dissimilarity structure of the data. Metric-preserving transformations, also known as isometries, of the hypersphere are given by orthogonal transformations. Therefore, the requirements for brain representation transfer led to an algorithm that distills the assumption of the commonality in similarity structure across participants into an isometric transformation across low-dimensional hyperspheres, using encoders for non-linear dimensional reduction implemented as a neural network. The isometric property used in the transformation would be useful for transferring brain representation not only between two participants but also across multiple participants.

### 2.2 Algorithm implementation for representation transfer

By combining several algorithms, including those selected in the previous section, we implemented a method for brain representation transfer that comprised three major computational steps:

(A) Embedding brain information into hyperspheres using encoders individually trained by the instance learning rule
(B) Aligning embedded brain information using an orthogonal matrix obtained through unsupervised object matching
(C) Fine-tuning encoders for improving the accuracy of the representation transfer

**FIGURE 1:**
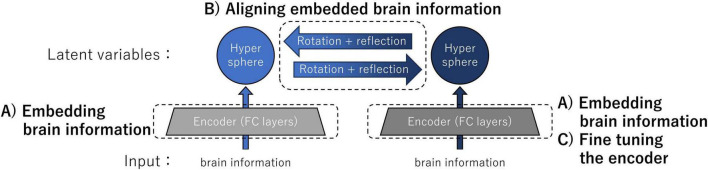
Overview of the architecture and computational steps for brain representation transfer. The latent variables were neural information embedded on the hypersphere using encoders individually trained by the instance learning rule. Representation transfer between latent variables was achieved by rotating and reflecting the embedded brain information on the hyperspheres. For improving the accuracy of the representation transfer, we performed fine-tuning of the encoder. Specific details of the three major computational steps A, B, and C are provided in the main text.

Figure 1 shows an overview of the architecture and computational steps of our transfer method implemented for the validation experiments. The inputs of individual encoders were neural information: in the validation experiments, the inputs consisted of either the intermediate-layer outputs of ANNs or the data derived from the fMRI responses in human participants. The neural information was embedded as latent variables on a hypersphere by individual encoders composed of two non-linear fully connected (FC) layers trained with the instance learning rule (computational step A). By determining the correspondence of latent variables using kernelized sorting, we estimated the orthogonal (rotation + reflection) matrix for aligning embedded brain information (computational step B). Finally, one of the encoders was fine-tuned by aligning the data distributions on the two hyperspheres (computational step C). This architecture enabled the exchange of latent variables, which are conceptual brain data that represent the sensations of individuals.

#### 2.2.1 Embedding brain information into hyperspheres using encoders individually trained by instance learning rule

We selected an n-dimensional hypersphere as the embedding space. We set n-dimension as 32-dimension for this experiment. The encoder comprised two layers: a non-linear FC layer with batch normalization and a rectified linear unit (ReLU) as an activation function; and an FC layer with L2 normalization. We set the dimensions of each layer in the encoder to be logarithmically spaced for the input, intermediate, and output layers. We trained the encoder using instance learning loss with a hyperparameter τ, which adjusts the repulsion between the positive and negative samples.

As a data augmentation, we added Gaussian noise to each dimension of input data, varying the noise with each input and iteration. Gaussian noise was generated by drawing multi-dimensional random numbers from a normal distribution with zero mean and standard deviations (SDs) of *k*_σ_⋅σ, where *k*_σ_ is a noise gain parameter and σ is the SD of each dimension of the training dataset.

To determine the hyperparameters, we performed a grid search for the repulsion parameter τ and the noise gain parameter *k*_σ_ (results of the grid search are available in the Supplementary materials). We determined the selected hyperparameter values to satisfy the following two conditions: First, the optimal parameters must maximize the skewness of the pairwise cosine similarity distribution of the training data embedded in a hypersphere. Second, the embedded data must become linearly independent within the range of machine precision when subjected to singular value decomposition. Empirically, we observed that varying the hyperparameter τ led to diverse distributions, where data points were either gathered at a single point on the hypersphere, dispersed uniformly, or formed clustered structures among similar data. It is more advantageous for the embedded data to have a clustered structure than to be uniformly dispersed when detecting distinctive data points in subsequent computational steps. Therefore, we used skewness as an index of cluster cohesiveness. Additionally, we found that setting τ and *k*_σ_ too large resulted in linear dependence among the latent variables, which indicated that the embedding space was not fully exploited, and the latent variables could be represented in a lower-dimensional space. Given these considerations, we determined the optimal values of the hyperparameters τ and *k*_σ_ using the skewness of the distribution and the linear independence of the embedded data as criteria.

#### 2.2.2 Aligning embedded brain information using an orthogonal matrix obtained through unsupervised object matching

The issue of finding corresponding points between datasets without using label information is known as the object-matching problem (Yamada and Sugiyama, 2011). Specifically, seeking one-to-one correspondence with a specific objective function is referred to as the quadratic assignment problem (Loiola et al., 2007). We used kernelized sorting (Quadrianto et al., 2010), which addresses the quadratic assignment problem by leveraging the similarity and dissimilarity relationships within the data. The kernelized sorting algorithm iteratively adjusts the order of samples in one dataset to align with those in another, with the aim of maximizing the value of the Hilbert–Schmidt Independence Criterion (HSIC). The objective function that the kernelized sorting aims to maximize is given by the following formula:


(1) $$\text{HSIC}\,\left(\pi ,\bar{K},\bar{L}\right)=\text{trace}\,\left(\bar{K}\,\pi^{T}\,\bar{L}\,\pi \right).$$

Here, $\bar{K}$ and $\bar{L}$ are the pairwise distance matrices with zero mean, which are calculated from data points within the datasets for participants A and B, respectively. Additionally, π represents a permutation matrix, and the HSIC value is calculated by permuting the order of the data points by π. Because of the possibility of encountering local optima, we performed multiple random shuffles of the initial order and selected the permutation with the highest HSIC value. As indicated in the above formula, HSIC evaluates the degree of correspondence between data samples based on the similar and dissimilar relationships within the dataset.

To apply kernelized sorting to the actual data, the number of data samples must match between the two datasets. Furthermore, given that searching through all of the training data are computationally expensive and impractical because there are *n!* different assignments for *n* samples, we reduced the computational cost of searching for corresponding points by selecting data according to cohesion. When the hyperparameters are set in a specific range during instance learning, the clusters form among similar embedded data points after instance learning. To extract these clusters, we calculated pairwise cosine distances between data points in the hypersphere and counted the number of neighbors with distances below a specific threshold for each data point. We considered data with many neighbors as representative points for clusters.

We determined the orthogonal matrix *R* that best aligned the matched representative points obtained through kernelized sorting: let matrices *P* and *Q* be vertically stacked representative points as row vectors for participants A and B, respectively. The orthogonal matrix *R* for transforming the representation from participant B to participant A can be obtained as *R* = *UV^T^* using singular value decomposition *Q^T^P* = *U*Σ*V^T^*, where *U* and *V* are orthogonal matrices, and Σ is a square diagonal matrix with singular values on the diagonal. We performed kernelized sorting and obtained the orthogonal matrix *R* for each participant pair.

#### 2.2.3 Fine-tuning encoders for improving the accuracy of the representation transfer

Although an orthogonal matrix enables overall alignment of the two datasets embedded on the surface of the hyperspheres, there is a possibility of persisting incomplete errors due to individual differences. To reduce such errors and improve the accuracy of brain representation transfer, we fine-tuned the encoder by aligning the data distributions on the two hyperspheres using maximum mean discrepancy (MMD) loss. Specifically, we kept one encoder (the FC layer network for participant A) fixed and additionally trained the other encoder (the FC layer network for participant B) using MMD loss to align its data distribution on the hypersphere with that of the fixed encoder.

### 2.3 Validation experiment conditions using ANNs

#### 2.3.1 Datasets

We generated datasets for brain representation transfer using intermediate-layer outputs of the ANNs as a substitute for neural activities of visual cortex. Some studies have demonstrated the similarity of information representation between the brain and ANNs based on the analysis utilizing the representational dissimilarity matrix (RDM). For example, studies have compared representation similarities between hierarchically organized brain regions in the human visual cortex and intermediate layers of an ANN (Kriegeskorte et al., 2008a; Hiramatsu et al., 2011; Cichy et al., 2016). In addition, similarities between optimal stimuli that evoke responses in neural cells and optimal stimuli that activate convolutional kernels in ANNs have been reported (Connolly et al., 2012). Therefore, we first conducted validation experiments of brain representation transfer using data acquired from ANNs, which allowed us to control various experimental factors.

We used ResNet20 (He et al., 2016), which was trained to classify images from the CIFAR-10 dataset (Krizhevsky, 2009) into 10 classes, using an unofficial implementation available at https://github.com/chenyaofo/pytorch-cifar-models. We randomly divided the 50,000 CIFAR-10 training images into two sets. We then independently trained the ResNet20 models on each set to create a condition in which no intermediate-layer output data in response to common images was available as training datasets to achieve representation transfer between two ANNs. We set the learning rate to 0.01, the batch size to 512 samples, the number of epochs to 5,000, and the number of negative samples for each positive sample to 4,096. To validate the proposed transfer method for multiple ANN pairs, we repeated this training process with nine different random seeds to split the CIFAR-10 training images into two sets. In addition to the training datasets, we acquired the intermediate-layer outputs from the paired trained ResNet20 models in response to the same set of 10,000 images as test datasets for the purpose of evaluation.

We randomly sampled the intermediate-layer outputs to duplicate the scenario whereby only partial data of a brain region was available through actual neural recordings. ResNet20 consists of approximately five blocks composed sequentially from the input side: one convolutional layer, three residual blocks, and one FC layer. In this study, we obtained the 8,192-dimensional output data from the second residual block in response to CIFAR-10 images. We then prepared the datasets by randomly sampling the output into 4,096 (50%), 2,048 (25%), and 1,024 (12.5%) dimensions. The same set of artificial neurons randomly selected from the intermediate layer was used to obtain both the training and test data.

#### 2.3.2 Model parameters

For the validation experiments of the brain representation transfer for ANNs, we projected each randomly sampled dataset onto a hypersphere in a 32-dimensional space using an encoder trained with the instance learning rule. The dimensions of the FC layers in the encoder were logarithmically spaced according to the dimensions of the datasets: 4,096 to 362 to 32 dimensions, 2,048 to 256 to 32 dimensions, and 1,024 to 181 to 32 dimensions, respectively. Based on the grid search results performed in advance (details in the Supplementary materials), we set the parameters (τ, *k*_σ_) for the instance learning to (0.8, 1.5) for 4,096 dimensions, (0.5, 1.5) for 2,048 dimensions, and (0.8, 0.75) for 1,024 dimensions, respectively.

We selected the top 1,600 representative data points from the embedded latent variables from the data of each ANN for kernelized sorting. The threshold for the cosine distance of training data pairs, which was used to count the number of neighbors and identify representative points for clusters, was set to 0.2. To prevent the selection of similar representative points, we selected only data with a cosine distance of a minimum of 0.2 from the already-selected representative points. We performed kernelized sorting 26,000 times for each ANN pair to obtain an orthogonal matrix with a plausible permutation that maximized the HSIC (Equation 1).

#### 2.3.3 Evaluation of noise tolerance obtained through instance learning

To investigate the noise tolerance obtained through instance learning, we examined the fluctuations in latent variables derived from input noise. First, we trained two encoders using instance learning: one with and one without the use of Gaussian perturbation as data augmentation (note: *E_N_* and *E_0_* are the projection of the encoders trained with and without Gaussian perturbation, respectively). Next, we obtained the latent variables for each encoder and calculated the cosine distance between the latent variables before and after noise was added to the test data. Let ${\tilde{x}}_{i}$ be *i*th test data *x_i_* with Gaussian noise ε, ${\tilde{x}}_{i}=x_{i}+\varepsilon$; let ${\tilde{y}}_{i}$ and *y_i_* be the embedded latent variables of ${\tilde{x}}_{i}$ and *x_i_* by the encoder, respectively. Therefore, the cosine distance *d_i_* between the latent variables before and after adding noise can be written as:


(2) $$d_{i}\,\left(y_{i},{\tilde{y}}_{i}\right)=1-\frac{y_{i}\cdot {\tilde{y}}_{i}}{||y_{i}||\,||{\tilde{y}}_{i}||},$$

$$\text{where}\,y_{i}=E\,\left(x_{i}\right),{\tilde{y}}_{i}=E\,\left({\tilde{x}}_{i}\right),E\in \left\{E_{0},E_{N}\right\}.$$

If the encoder is robust to input noise, *d_i_* will be close to zero. We embedded a 4,096-dimensional ANN dataset, obtained from ResNet20 as a model of the visual cortex, into latent variables on a hypersphere. We generated 50 different ${\tilde{x}}_{i}$ with varying noise patterns and calculated *d_i_* for all test data.

#### 2.3.4 Quantitative evaluation of representation transfer

If the proposed method of brain representation transfer is successful, the brain information transferred from an independent ANN (or participant) should match that we obtained ourselves. To quantitatively evaluate our method, we calculated the cosine similarities between the embedded latent variables from the test data of two ANNs with corresponding index labels. We defined the averaged cosine similarity for test data as the “alignment score,” which was calculated using the following formula:


(3) $$\mathrm{alignment}\,\mathrm{score}=\sum_{i}\frac{y_{i}\cdot R\,{\hat{y}}_{i}}{||y_{i}||\,||R\,{\hat{y}}_{i}||},$$

$$\text{where}\,y_{i}=E_{A}\,\left(x_{i}\right),{\hat{y}}_{i}=E_{B}\,\left(x_{i}\right).$$

In this formula, where, *y_i_* and ${\hat{y}}_{i}$ are *i*th latent variables encoded with *i*th input data, *x_i_* and ${\hat{x}}_{i}$, derived from ANN A and B, respectively. *R* denotes the orthogonal matrix obtained for the ANN pair, which transforms the brain representation from ANN B to ANN A. Because the orthogonal matrix *R* is reversible, the alignment scores calculated in the latent space for ANN A and ANN B are identical. Furthermore, to obtain the upper bound for each transfer task, we calculated the alignment score using the orthogonal matrix determined from the correspondence of the test data. The defined evaluation method was also applied to the data obtained from participants. The alignment scores are presented in arbitrary units.

#### 2.3.5 Image category discrimination of the latent variables

To quantitatively assess whether conceptual information can be transmitted across ANNs, we conducted image category discrimination in latent space. The CIFAR-10 dataset has 10 types of category labels assigned to images. The ResNet20 Layer2 outputs used for inter-ANN representation transfer exhibited similar outputs for the same category; moreover, the latent variables in the embedded hypersphere formed a clustered structure based on categories. We assigned the estimated category of test data based on the mode of the category labels among its 20-nearest neighbors from the training data in latent space.

In the verification experiments, we conducted both intra- and inter-ANN image category discrimination. For intra-ANN discrimination, we calculated the accuracy of image category discrimination according to the nearest neighbors of the test and train latent variables from the same ANN. For inter-ANN discrimination, accuracy was calculated using the test latent variables transferred from another ANN and the train latent variables of one ANN.

#### 2.3.6 Image reconstruction from latent variables

To qualitatively assess whether conceptual information can be transmitted across ANNs, we reconstructed the input images from the latent variables and applied a super-resolution technique to the reconstructed images using a diffusion model (Sohl-Dickstein et al., 2015; Ho et al., 2020; Kingma et al., 2021). The structure of the decoder was similar to that of the encoder and comprised two FC layers with batch normalization and ReLU activation, followed by three deconvolution layers (see Supplementary materials for further details). We trained the decoder until the mean squared error (pixel loss) between the input images and reconstructed images became sufficiently small, while fixing the weights of the ResNet20 model and encoder. In addition, we applied Gaussian noise to the inputs of the FC layers for data augmentation after random sampling, similar to the procedure used to train the encoder. The decoder was trained using the image set that was used to train the aforementioned single ANN. The test images used for evaluation in the main experiment were not included in this training set.

The diffusion model for super-resolution refinement of the reconstructed images used an unofficial PyTorch implementation of image super-resolution via repeated refinement (SR3) (Saharia et al., 2023).^1^ We trained the model to conditionally generate 32 × 32-pixel images from reconstructed images of the same size, based on a script provided for FFHQ images (Karras et al., 2018), which were originally designed for conditional generation with super-resolution from 16 × 16 to 128 × 128 (see Supplementary materials for further details). The diffusion model was trained using the same image set as the decoder, and again test images were not included.

### 2.4 Validation experiment conditions using data from human participants

#### 2.4.1 Datasets

As another verification experiment of our proposed method, we used datasets of blood oxygen level-dependent (BOLD) signals evoked by watching movies acquired using fMRI. Specifically, we used BOLD response patterns associated with concepts of multiple words, as reported previously (Nishida et al., 2020; Matsumoto et al., 2023). Henceforth, we will refer to such brain activity associated with the concepts of words in humans as “brain word representation.” Data were acquired from seven healthy participants using the calculation method developed by Nishida et al. (2020).

The method for calculating brain word representations was as follows. First, we used a pre-trained word2vec model (Mikolov et al., 2013) to convert scene descriptions for each second of the movie into semantic vectors within the word2vec vector space. We then determined a weight matrix of a regression model for each participant, which estimated the BOLD signal of each voxel from the semantic vectors of the scenes. We accounted for a delay of 2, 4, and 6 s following the scene presentation for the BOLD signal evoked by the movie scenes. Finally, we obtained participant-specific brain word representations by calculating the product of the weight matrix and word vectors among frequently occurring words (limited to nouns, verbs, and adjectives) in the Japanese Wikipedia. Words and training data for the word2vec model were obtained from the Japanese Wikipedia corpus, dumped on 11 January 2016.

We randomly divided the brain word representations of the 30,000 most frequently occurring words in the Japanese Wikipedia into training data (25,000 words) and test data (5,000 words). Because we selected the 1,400 voxels with the highest predictive performance of the BOLD signal during weight matrix estimation, the brain word representations resulted in 4,200 dimensions (for the three delays for each voxel). The label information corresponding to the brain word representation was consistent across participants. However, corresponding label information was used only for the performance evaluation of the transfer task and subsequent confirmation of the representational similarities based on RDMs: the training process of brain representation transfer was conducted without label information.

The calculation methods for the alignment score and quantitative evaluation metric for the proposed method using test data were the same as those used for inter-ANN brain representation transfer.

#### 2.4.2 Model parameters

For the validation of brain representation transfer of human fMRI responses, we projected the participant-specific 4,200-dimensional brain word representation dataset onto a hypersphere in the 32-dimensional space. We set the dimensions of the FC layers in the encoder to be logarithmically spaced: 4,200 to 367 to 32 dimensions. Based on the grid search results, we set the parameters (τ, *k*_σ_) for each participant in instance learning, where the skewness of the pairwise cosine similarity distribution of the latent variables was maximized to ensure that the latent variables were linearly independent. The value of τ ranged from 0.4 to 0.6, and that of *k*_σ_ ranged from 1.5 to 2.5. Similar to the validation experiment using ANNs, we set the number of representative data points of clusters to 1,600 for kernelized sorting and the threshold for choosing the representative points to 0.1 in cosine distance measure. We performed kernelized sorting 1,000 times for each participant pair to obtain the orthogonal matrix with a plausible permutation that maximized the HSIC.

#### 2.4.3 Quantitative evaluation of representation transfer

Similar to the validation experiments using ANNs, we calculated the alignment score to evaluate the performance of representation transfer (see Section “2.3.4 Quantitative evaluation of representation transfer”). Furthermore, to investigate the relationship between alignment performance and the commonality of representation similarities in datasets across participants, we analyzed the correlation between the alignment score and the correlation coefficient of the RDMs between the two datasets.

We calculated the RDMs of the test datasets in two ways: (1) the RDM defined as the pairwise Euclidian distance of the brain word representation datasets in a 4,200-dimensional Euclidian space; and (2) the RDM defined as the pairwise cosine distance of the latent variables of the test datasets embedded by the trained encoder. The calculation of the RDM, however, requires the corresponding labels between the two datasets.

As an index to evaluate the commonality of representation similarities in datasets across participants without the use of preassigned correspondence labels, we defined a normalized HSIC value given by the following formula:


(4) $$\mathrm{normalized}\,\mathrm{HSIC}\,\left(\pi ,\bar{K},\bar{L}\right)=\frac{\text{trace}\,\left(\bar{K}\,\pi^{T}\,\bar{L}\,\pi \right)}{\sqrt{\text{trace}\,\left({\bar{K}}^{T}\,\bar{K}\right)}\,\sqrt{\text{trace}\,\left({\bar{L}}^{T}\,\bar{L}\right)}}.$$

Here, π, $\bar{K}$, and $\bar{L}$ are defined in Section “2.3.2 Model parameters.” We used this normalized version of HSIC because the range of the HSIC value depends on $\bar{K}$ and $\bar{L}$, which makes it difficult to compare between different participant pairs. We derived this formula based on the analogy between covariance and correlation. We calculated the correlation coefficient between the alignment scores and the highest normalized HSIC values obtained from kernelized sorting for multiple participant pairs. HSIC and normalized HSIC values are presented in arbitrary units.

## 3 Results

### 3.1 Noise robustness through data augmentation during instance learning

Neural activity data typically contain fluctuations in the recorded system and noise from measurement instruments. Therefore, ensuring the proper embedding of obtained data into latent variables, regardless of these fluctuations and noise, while preserving the inherent similarity structure, is critical for achieving successful brain representation transfer in downstream processing. We investigated the differences in noise resilience obtained through instance learning with and without the addition of Gaussian noise as data augmentation using ANN datasets (synthetic datasets prepared in Section “2.3.1 Datasets”).

**FIGURE 2:**
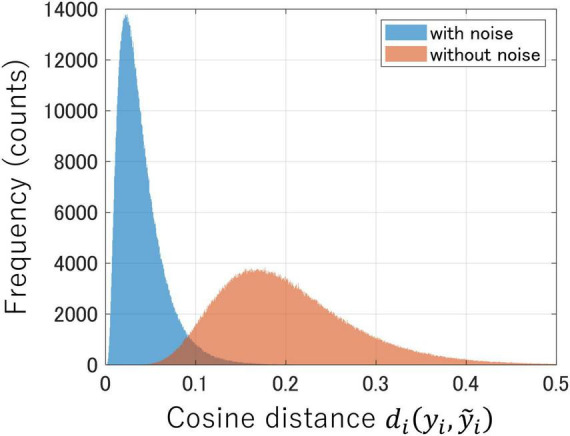
Comparison of noise robustness between encoders trained with Gaussian noise (blue) and the one trained without noise (red). Each distribution represents the cosine distances between latent variables before and after noise was added to the test data.

Figure 2 shows the distributions of the cosine distances between latent variables before and after adding Gaussian noise to the test data (Equation 2) in both encoders trained with and without noise. If the encoder is sufficiently robust to noise, the distribution of cosine distances will converge to zero. In Figure 2, the encoder trained with Gaussian noise as data augmentation (depicted in blue) had a distribution near zero, which indicated reduced variability in the latent space against noise perturbation to the test data. In contrast, the encoder trained without noise (depicted in red) exhibited a distribution further away from zero, which suggested greater variability in the latent space owing to the influence of input noise. Therefore, data augmentation with Gaussian noise during instance learning enhances the noise robustness of the encoder.

### 3.2 Inter-ANN representation transfer

**FIGURE 3:**
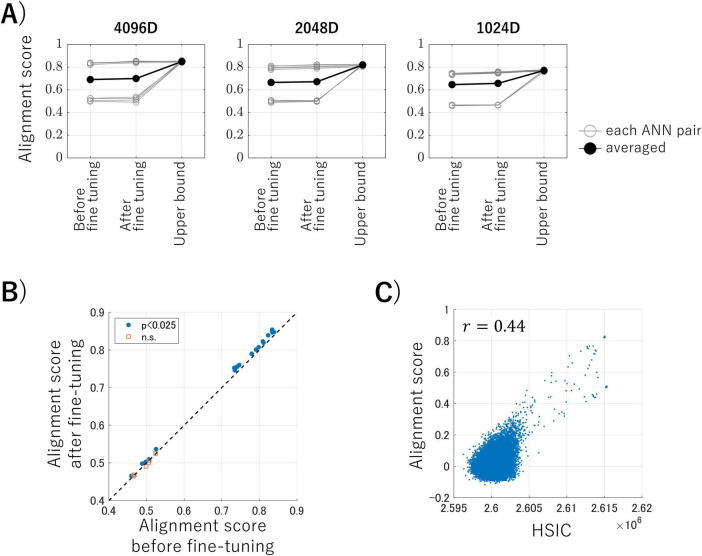
**(A)** Alignment scores for each dataset under three conditions. Each panel represents the results for the datasets obtained by randomly sampling artificial neural network (ANN) outputs into 4,096 dimensions (50% of the total), 2,048 dimensions (25%), and 1,024 dimensions (12.5%), respectively. Each gray line in the panels represents one ANN pair, and the black lines represent the averaged alignment scores for each condition. **(B)** The results of the one-sided paired *t*-tests before and after fine-tuning. Blue dots indicate pairs that showed a significant improvement in alignment score after fine-tuning the encoder, and the red squares represent pairs that showed no significant improvement (*p* < 0.025, Bonferroni corrected). **(C)** An example of the relationship between the alignment scores and Hilbert–Schmidt Independence Criterion (HSIC) values for each kernelized sorting result.

We conducted brain representation transfer using the intermediate-layer outputs of ANNs as activity of artificial neurons, which allowed us to control various experimental factors. Figure 3A shows the alignment scores (Equation 3), which are the averaged cosine similarities between the latent variables of the test data, for brain representation transfer under three conditions: using encoders before fine-tuning, using encoders after fine-tuning with MMD loss (the proposed method), and using transformation obtained with the correspondence information of test data (serving as the upper bounds of this transfer task). Each panel represents the results of the datasets obtained by randomly sampling ANN outputs into 4,096 dimensions (50% of the total), 2,048 dimensions (25%), and 1,024 dimensions (12.5%). Each gray line in the panels represents one ANN pair, and the black lines represent the averaged alignment scores for each condition. These results suggest that our proposed method, in which no correspondence labels across ANNs are available, achieves alignment scores close to those obtained when using correspondence labels. Surprisingly, the proposed method achieved this performance even with embeddings from a limited 1,024-dimensional data (i.e., 12.5% of the total data).

We conducted statistical analyses to determine whether the alignment score could be improved by fine-tuning the encoder during the third computational step of the proposed algorithm during inter-ANN representation transfer. Figure 3B shows the results of the one-sided paired *t*-tests for each ANN pair on the inter-ANN cosine similarities of the latent variables in response to test data (10,000 samples), before and after fine-tuning. ANN pairs trained with nine different random seeds and three different rates of output sampling result in 27 pairs for comparison. The blue dots indicate pairs in which the alignment score was significantly improved following fine-tuning of the encoder, whereas the red squares signify pairs that showed no significant improvement (degree of freedom = 9999, *p* < 0.025, Bonferroni corrected). These results suggest that the alignment scores of ANN pairs with high alignment scores can be improved through fine-tuning the encoder using MMD loss.

Some ANN pairs had low alignment scores that did not reach the upper bound. Figure 3C illustrates the relationship between the alignment scores and the HSIC values for each kernelized sorting result in an example ANN pair. There was a positive correlation (*r* = 0.44) between the alignment scores and the HSIC values, which confirmed that the HSIC serves as a satisfactory criterion for unsupervised object matching using kernelized sorting. However, the highest HSIC value did not consistently result in a high alignment score, which indicated that the results of the unsupervised object matching for ANN data were prone to local optima rather than global optima. This trend was also observed in other ANN pairs (Supplementary Figure 3).

As an alternative evaluation metric for brain representation transfer, we conducted a 10-category discrimination of the latent variables using the category labels assigned to images. For the baseline, the averaged accuracy for the discrimination task within the ANN (i.e., without brain representation transfer) was 63.21% (SD = 0.62%), 62.15% (SD = 0.61%), and 60.38% (SD = 0.59%), for the 4,096-dimensional, 2,048-dimensional, and 1,024-dimensional datasets, respectively. In contrast, the averaged accuracy for the discrimination task with inter-ANN transfer was 60.38% (SD = 0.83%), 59.07% (SD = 0.69%), and 55.11% (SD = 0.71%) for the 4,096-dimensional, 2,048-dimensional, and 1,024-dimensional datasets, respectively. These results suggest that the proposed method can consistently transfer category information between ANNs.

**FIGURE 4:**
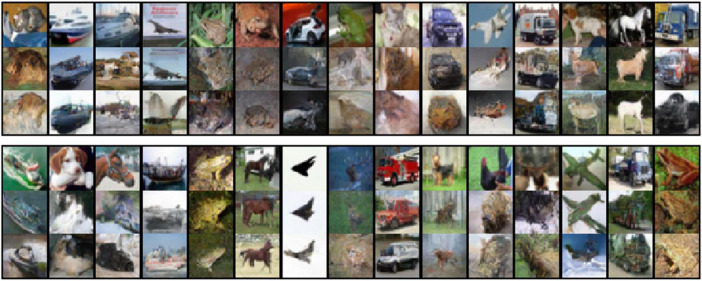
Test and reconstructed images from the latent variables. The results for different examples are shown in two panels. In each panel, the top row of tiled images represents the test images inputted into the artificial neural networks (ANNs). The middle row represents the reconstructed images from the latent variables without representation transfer and shows the baseline performance of the decoding model. The bottom row represents the reconstructed images with inter-ANN representation transfer.

To demonstrate that our proposed algorithm is compatible with machine learning techniques and satisfies requirement (iii), we conducted image reconstruction from the latent space and performed super-resolution refinement using a diffusion model. We pre-trained the decoder for one of nine ANNs and the diffusion model using a 4,096-dimensional dataset obtained using randomly sampled outputs of the intermediate layer of ResNet20. Figure 4 shows the original images inputted into the ANN (top row), reconstructed images from the latent variables of the ANN without representation transfer (middle row), and the reconstructed images with inter-ANN representation transfer (bottom row). When the middle and bottom rows of Figure 4 were compared, we observed that the reconstructed images were qualitatively similar. This indicates that a decoding model personalized to one ANN can also accurately decode latent information from another ANN using our proposed representation transfer method.

### 3.3 Inter-participant representation transfer

**FIGURE 5:**
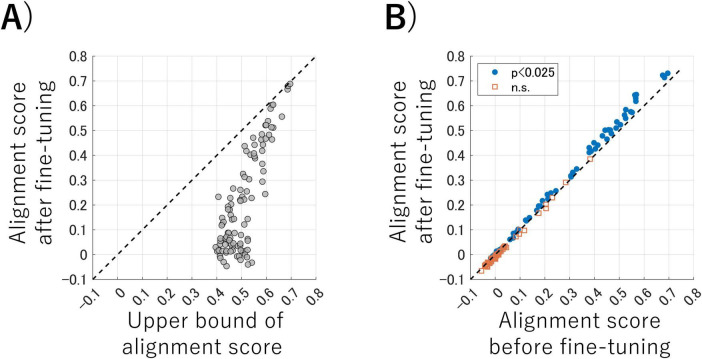
**(A)** Comparison of alignment scores between the proposed method and the upper bound of this transfer task derived from the correspondence information of the test data. Each dot represents each participant pair. **(B)** The results of the one-sided paired *t*-test before and after fine-tuning. Blue dots represent pairs that showed significant improvement in alignment score after fine-tuning the encoder, whereas red squares represent pairs that showed no significant improvement (*p* < 0.025, Bonferroni corrected).

In this experiment, we used the brain word representations derived from the fMRI responses of human participants instead of ANN model data to conduct brain representation transfer using our proposed algorithm. Figure 5A shows the comparison between the alignment scores obtained using our method and the upper bound of this transfer task derived from the correspondence information of the test data. Each dot represents each participant pair. The alignment scores of our proposed method approached the upper bound, even without the use of corresponding labels for several pairs of participants. Conversely, although the alignment scores could exceed 0.4 when correspondence information was available, we also observed that the alignment scores using our method were near zero for some participant pairs.

We statistically examined whether fine-tuning the encoder using MMD loss during the third computational step of the proposed algorithm improved alignment scores to facilitate the overall matching of latent variables. First, we performed a two-way analysis of variance of the inter-participant cosine similarities of the latent variables in response to test data (5,000 samples) for 21 participant pairs with five replications of instance learning. The statistical factors were before and after encoder fine-tuning and the 21 participant pairs. We revealed significant main effects for both factors (Main effect of participant pair: F = 26762.35, degree of freedom = 20, *p* < 0.001. Main effect of fine-tuning: F = 587.96, degree of freedom = 1, *p* < 0.001). We then performed one-sided paired *t*-tests for each participant pair for the inter-participant cosine similarities of the latent variables in response to test data (5,000 samples), with before and after fine-tuning as factors. The results of the one-sided paired *t*-tests are shown in Figure 5B. 21 participant pairs with five replications of instance learning result in 105 pairs for comparisons. The blue dots represent pairs that showed significant improvement in alignment score after fine-tuning the encoder, whereas the red squares represent pairs that showed no significant improvement (degree of freedom = 4999, *p* < 0.025, Bonferroni corrected). These findings suggest that fine-tuning the encoder resulted in a significant improvement in the alignment scores of participant pairs with high alignment scores.

The performance of the proposed representation transfer method showed large variation across participant pairs in the previous analysis. To examine the factors contributing to this variability in transfer performance, we assessed the representational similarity between participants using their corresponding labels. We found that participant pairs with low alignment scores also exhibited low representational similarity, as assessed by the correlation coefficients between RDMs. When we used RDMs derived from the pairwise Euclidean distances of the brain word representations, the correlation coefficients between alignment scores and RDM similarities for all participant pairs was 0.725 (Supplementary Figure 8A). Similarly, when using RDMs derived from pairwise cosine distances of latent variables, the correlation coefficient was 0.774 (Supplementary Figure 8B). We observed high correlations in both cases, which indicated that our proposed method was effective as long as the data satisfied the basic assumption of commonality in representational similarity across participants. Moreover, our results suggest that if representational similarities between participants can be predicted in advance using an unsupervised method, alignment scores can also be predicted.

**FIGURE 6:**
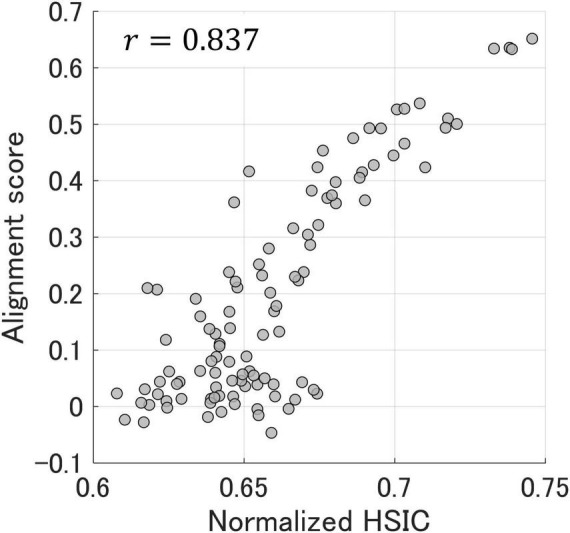
The relationship between alignment scores and normalized HSIC values obtained from plausible matching through kernelized sorting. Each dot represents one participant pair. The correlation coefficient between the alignment scores and normalized HSIC values was 0.837.

Figure 6 shows the relationship between the alignment scores and normalized HSIC values (Equation 4) obtained from the plausible matching through kernelized sorting, and the correlation coefficient was 0.837. It is noteworthy that the HSIC could be calculated by a certain permutation using kernelized sorting without the use of preassigned corresponding labels. This result indicates that a normalized HSIC optimized by kernelized sorting allows unsupervised prediction of alignment scores and representational similarities among participants.

## 4 Discussion

We proposed a method for brain representation transfer, whereby data representation obtained from one person’s brain can be transformed to another, without the use of corresponding label information between the two datasets. The proposed method operates under the assumption that the similar and dissimilar relationships in the latent properties of the data are common across participants. To validate our proposed method, we conducted two experiments: in one experiment, we used the intermediate-layer outputs of ANNs in response to various images as a substitute for neural activity in the visual cortex, and in the second experiment, we used data derived from fMRI activity of human participants (i.e., brain word representation).

The validation experiment using ANNs demonstrated that our method can successfully perform representation transfer and achieve high alignment scores; in some cases, these scores were similar to those obtained when using corresponding labels. Additionally, quantitative evaluation using image category discrimination in latent space suggested that conceptual information can be transmitted across ANNs. Moreover, we showed that images can be reconstructed from latent variables after inter-ANN representation transfer with a quality similar to images reconstructed without performing representation transfer. Our result demonstrates the possibility that images can be reconstructed from an individual’s brain data without training personalized decoders through the use of brain representation transfer.

The validation experiment using human fMRI data revealed that the alignment score using our method is dependent on the similarity of data representation between participants. The alignment score was high—in some cases, it reached its upper bound—when our algorithm was able to exploit a common representation structure across participants. However, the alignment score dropped dramatically when the difference in data representation between two participants was too large to extract a common latent property in the absence of preassigned corresponding label information. We demonstrated that a normalized HSIC optimized by kernelized sorting is a useful measure for predicting representational similarity between participants without the use of corresponding labels. Therefore, this measure would be valuable for assessing, in advance, whether our proposed method can successfully perform representation transfer for a given participant pair.

In this study, we performed brain representation transfer exclusively on datasets related to visual information using artificial neuronal activity or human brain activity. However, theoretically, the proposed method would apply not only to visual information but also to other modalities, such as auditory or somatosensory information. This is because our method relies on the general assumption that the similar and dissimilar relationships in the latent properties of the data exhibit certain commonalities across participants.

In the validation experiment of inter-ANN representation transfer, although the alignment scores were close to the upper bound in some ANN pairs, the other pairs yielded marginal scores. As described in Section “3 Results”, the ANN datasets were prone to local optima during the unsupervised object matching using kernelized sorting. Indeed, the latent variables were strongly clustered around each category, to the extent that the image categories of the test data could be predicted by the k-nearest neighbors algorithm. When conducting kernelized sorting on data with a sparse distribution, the cost function tends to fluctuate significantly with minor changes in the plausible matching, which often results in a tendency to remain trapped in local optima. In the future, we plan to investigate data structures and develop embedding techniques for data that are less susceptible to local optima.

In the validation experiment of inter-participant representation transfer, we used datasets comprising brain word representations, which were estimated BOLD signals representing the concepts of various words, instead of simply employing BOLD signals evoked by visual stimuli. This is because the raw BOLD signals exhibited large individual and trial variation, which hindered the extraction of common similar and dissimilar relationships across participants for our proposed transfer method. We also observed low representational similarities, as assessed by the correlation coefficient between RDMs calculated from the raw BOLD signal without pre-processing or normalization. The brain word representations used in this study serve as an example of preprocessing that ensures representational similarity between participants for our proposed transfer method. Alternative effective preprocessing methods that allow representation transfer warrant further investigation.

We constructed the entire brain representation transfer algorithm by combining several algorithms that satisfy the following three requirements: (i) robust dimensionality reduction, preserving similarity and dissimilarity structures while managing various types of noise and individual differences; (ii) reversible transformation ensuring cycle consistency; and (iii) compatibility with machine learning techniques used for decoding brain information. As an algorithm that satisfies requirements (i) and (iii), we applied instance learning (Wu et al., 2018), which was originally proposed as a variant of contrastive learning in the field of computer vision. We demonstrated that adding Gaussian noise as a data augmentation method during the training phase contributes to noise resilience in the representation of latent variables following dimension reduction. Furthermore, we showed that images can be reconstructed from latent variables using a decoding model, which satisfied requirement (iii). Additionally, we applied an orthogonal transformation, as an algorithm that satisfies requirement (ii), and used kernelized sorting as an unsupervised object-matching algorithm to estimate the orthogonal matrices. This implementation exploited the ability of the instance learning algorithm to embed brain information onto a hypersphere. However, our implementation of the transfer algorithm can be further improved with regard to the modules and choice of algorithms, which we describe below.

We used the instance learning algorithm proposed by Wu et al. to embed brain information onto a hypersphere. However, the instance learning algorithm inherently attempts to uniformly distribute the input data on the hypersphere. This is disadvantageous for detecting distinctive data points to estimate the orthogonal matrix. The use of other embedding methods that maintain similar and dissimilar relationships, including other contrastive learning methods, may address this issue.

Although we used the kernelized sorting algorithm for unsupervised object matching, other algorithms could have been used. For example, there is an optimal transport algorithm based on Gromov-Wasserstein distance (Mémoli, 2011) that has been used to determine word correspondence across different languages from word similarity relationships (Alvarez-Melis and Jaakkola, 2018). Indeed, recent studies by Takeda et al. (2024) and Takahashi et al. (2024) have demonstrated that the Gromov-Wasserstein Optimal Transport method is effective in aligning the brain activity datasets obtained from different participant groups, as well as datasets from ANNs. Incorporating potentially superior unsupervised object-matching algorithms into our method to align representational spaces across participants for representation transfer is a promising approach we would like to explore in future work. The kernelized sorting algorithm used in our study was designed to identify one-to-one mapping. However, depending on the dataset characteristics and the selection method of the representative points for object matching, one-to-one associations between two datasets may not always exist. Modifying the algorithm to detect many-to-one associations may offer a better method than kernelized sorting for finding better correspondence.

There are also alternative methods for selecting representative points when using kernelized sorting for unsupervised object matching. For example, hierarchical clustering (Nielsen, 2016) allows the creation of a tree structure based on the similar and dissimilar relationships between data; moreover, a clustered structure grouping similar data can be obtained by setting a threshold. In addition, if the characteristics of the data are known in advance, the k-means method may be a suitable approach (Fix and Hodges, 1989; Cover and Hart, 1967), using the number of clusters as a parameter.

In order for the decoding model to reconstruct images from latent variables, we combined two separate models: one was a naïve decoder for image reconstruction that comprised FC and deconvolutional layers; the other was a diffusion model for super-resolution refinement. An alternative decoding model would be to directly input the latent variables as conditions into the diffusion process for image generation, such as a latent diffusion model (Rombach et al., 2022). A recent study has applied this architecture to the reconstruction of high-detail presentation images from human fMRI data (Takagi and Nishimoto, 2023).

Although our study was focused on transforming data representations obtained from one brain to another, advanced techniques for both the acquisition and manipulation of brain activity need to be further integrated into our algorithm to achieve direct brain-to-brain communication to transmit sensations, intentions, and various other types of information. Neural activity can be acquired using various recording methods: not only fMRI, local field potentials, and EEG (as outlined in the introduction) but also functional near-infrared spectroscopy, electrocorticogram, and calcium imaging. Furthermore, numerous studies have induced sensory loss or generated new sensations by forcibly deactivating or activating individual neurons or localized neural cell populations using various techniques, such as needle electrodes (Lewis and Rosenfeld, 2016), transcranial magnetic stimulation (Kammer et al., 2005), and focused ultrasound (Yoo et al., 2011). Combining such acquisition and manipulation techniques with our proposed representation transfer method could potentially achieve direct communication between brains.

## Data Availability

Publicly available datasets were analyzed in this study. Data reported in this manuscript are available from the corresponding author of Matsumoto et al. (2023) on reasonable request.

## Footnotes

1
https://github.com/janspiry/image-super-resolution-via-iterative-refinement

## References

[B1] Alvarez-Melis D. Jaakkola T. (2018). “Gromov-wasserstein alignment of word embedding spaces,” in *Proceedings of the 2018 Conference on Empirical Methods in Natural Language Processing*, (Association for Computational Linguistics), 1881–1890.

[B2] Carlson T. A. Schrater P. He S. (2003). Patterns of activity in the categorical representations of objects. *J. Cogn. Neurosc.* 15 704–717. 10.1162/089892903322307429 12965044

[B3] Chen T. Kornblith S. Norouzi M. Hinton G. (2020). A simple framework for contrastive learning of visual representations. *arxiv* [Preprint] 10.48550/arXiv.2002.05709

[B4] Cheng D. Qiu N. Zhao F. Mao Y. Li C. (2021). Research on the modality transfer method of brain imaging based on generative adversarial network. *Front. Neurosci.* 15:655019. 10.3389/fnins.2021.655019 33790739 PMC8005554

[B5] Cichy R. M. Khosla A. Pantazis D. Torralba A. Oliva A. (2016). Comparison of deep neural networks to spatio-temporal cortical dynamics of human visual object recognition reveals hierarchical correspondence. *Sci. Rep.* 6:27755. 10.1038/srep27755 27282108 PMC4901271

[B6] Connolly A. C. Guntupalli J. S. Gors J. Hanke M. Halchenko Y. O. Wu Y. (2012). The representation of biological classes in the human brain. *J. Neurosci.* 32 2608–2618. 22357845 10.1523/JNEUROSCI.5547-11.2012 PMC3532035

[B7] Cover T. Hart P. (1967). Nearest neighbor pattern classification. *IEEE Trans. Inf. Theory* 13 21–27. 10.1109/TIT.1967.1053964

[B8] Fishman R. S. (1997). Gordon holmes, the cortical retina, and the wounds of war. *Documenta Ophthalmol.* 93 9–28. 10.1007/BF02569044 9476602

[B9] Fix E. Hodges J. L. (1989). Discriminatory analysis. nonparametric discrimination: Consistency properties. *Int. Stat. Rev.* 57 238–247. 10.2307/1403797

[B10] Grau C. Ginhoux R. Riera A. Nguyen T. L. Chauvat H. Berg M. (2014). Conscious brain-to-brain communication in humans using non-invasive technologies. *PLoS One* 9:e105225. 10.1371/journal.pone.0105225 25137064 PMC4138179

[B11] Haxby J. V. Gobbini M. I. Furey M. L. Ishai A. Schouten J. L. Pietrini P. (2001). Distributed and overlapping representations of faces and objects in ventral temporal cortex. *Science* 293 2425–2430. 10.1126/science.1063736 11577229

[B12] Haxby J. V. Guntupalli J. S. Nastase S. A. Feilong M. (2020). Hyperalignment: Modeling shared information encoded in idiosyncratic cortical topographies. *ELife* 9:e56601. 10.7554/eLife.56601 32484439 PMC7266639

[B13] Hayashi R. Kawata H. (2018). “Image reconstruction from neural activity recorded from monkey inferior temporal cortex using generative adversarial networks,” in *Proceedings of the 2018 IEEE International Conference on Systems, Man, and Cybernetics (SMC)*, (Piscataway, NJ: IEEE), 105–109.

[B14] He K. Zhang X. Ren S. Sun J. (2016). “Deep residual learning for image recognition,” in *Proceedings of the 2016 IEEE Conference on Computer Vision and Pattern Recognition (CVPR), 770–778*, (Piscataway, NJ: IEEE).

[B15] Hiramatsu C. Goda N. Komatsu H. (2011). Transformation from image-based to perceptual representation of materials along the human ventral visual pathway. *NeuroImage* 57 482–494. 10.1016/j.neuroimage.2011.04.056 21569854

[B16] Ho J. Jain A. Abbeel P. (2020). Denoising diffusion probabilistic models. *arxiv* [Preprint] 10.48550/arXiv.2006.11239

[B17] Hotelling H. (1936). Relations between two sets of variates. *Biometrika* 28 321–377. 10.2307/2333955

[B18] Hubel D. H. Wiesel T. N. (1962). Receptive fields, binocular interaction and functional architecture in the cat’s visual cortex. *J. Physiol.* 160 106–152. 10.1113/jphysiol.1962.sp006837 14449617 PMC1359523

[B19] Jiang L. Stocco A. Losey D. M. Abernethy J. A. Prat C. S. Rao R. P. N. (2019). BrainNet: A multi-person brain-to-brain interface for direct collaboration between brains. *Sci. Rep.* 9:6115. 10.1038/s41598-019-41895-7 30992474 PMC6467884

[B20] Kamitani Y. Tong F. (2005). Decoding the visual and subjective contents of the human brain. *Nat. Neurosci.* 8 679–685. 10.1038/nn1444 15852014 PMC1808230

[B21] Kammer T. Puls K. Erb M. Grodd W. (2005). Transcranial magnetic stimulation in the visual system. II. Characterization of induced phosphenes and scotomas. *Exp. Brain Res.* 160 129–140. 10.1007/s00221-004-1992-0 15368087

[B22] Karras T. Laine S. Aila T. (2018). A style-based generator architecture for generative adversarial networks. *IEEE Trans. Pattern Anal. Mach. Intell.* 43 4217–4228. 10.1109/TPAMI.2020.2970919 32012000

[B23] Kingma D. P. Salimans T. Poole B. Ho J. (2021). Variational diffusion models. *arxiv* [Preprint] 10.48550/arXiv.2107.00630

[B24] Komatsu H. Ideura Y. Kaji S. Yamane S. (1992). Color selectivity of neurons in the inferior temporal cortex of the awake macaque monkey. *J. Neurosci.* 12 408–424. 1740688 10.1523/JNEUROSCI.12-02-00408.1992 PMC6575605

[B25] Kriegeskorte N. Mur M. Bandettini P. (2008a). Representational similarity analysis - connecting the branches of systems neuroscience. *Front. Syst. Neurosci.* 2:2. 10.3389/neuro.06.004.2008 19104670 PMC2605405

[B26] Kriegeskorte N. Mur M. Ruff D. A. Kiani R. Bodurka J. Esteky H. (2008b). Matching categorical object representations in inferior temporal cortex of man and monkey. *Neuron* 60 1126–1141. 10.1016/j.neuron.2008.10.043 19109916 PMC3143574

[B27] Krizhevsky A. (2009). *Learning Multiple Layers of Features from Tiny Images.* Technical Report. Available at: https://www.cs.toronto.edu/~kriz/learning-features-2009-TR.pdf

[B28] Kruskal J. B. (1964a). Nonmetric multidimensional scaling: A numerical method. *Psychometrika* 29 115–129. 10.1007/BF02289694

[B29] Kruskal J. B. (1964b). Multidimensional scaling by optimizing goodness of fit to a nonmetric hypothesis. *Psychometrika* 29 1–27. 10.1007/BF02289565

[B30] Lee W. Kim S. Kim B. Lee C. Chung Y. A. Kim L. (2017). Non-invasive transmission of sensorimotor information in humans using an EEG/focused ultrasound brain-to-brain interface. *PLoS One* 12:e0178476. 10.1371/journal.pone.0178476 28598972 PMC5466306

[B31] Lewis P. M. Rosenfeld J. V. (2016). Electrical stimulation of the brain and the development of cortical visual prostheses: An historical perspective. *Brain Res.* 1630 208–224. 10.1016/j.brainres.2015.08.038 26348986

[B32] Liu H. Agam Y. Madsen J. R. Kreiman G. (2009). Timing, timing, timing: Fast decoding of object information from intracranial field potentials in human visual cortex. *Neuron* 62 281–290. 10.1016/j.neuron.2009.02.025 19409272 PMC2921507

[B33] Loiola E. M. de Abreu N. M. M. Boaventura-Netto P. O. Hahn P. Querido T. (2007). A survey for the quadratic assignment problem. *Eur. J. Oper. Res*. 176, 657–690.

[B34] Maaten L. Hinton G. (2008). Visualizing data using t-SNE. *J. Machine Learn. Res.* 9 2579–2605.

[B35] Matsumoto Y. Nishida S. Hayashi R. Son S. Murakami A. Yoshikawa N. (2023). Disorganization of semantic brain networks in schizophrenia revealed by fMRI. *Schizophrenia Bull.* 49 498–506. 10.1093/schbul/sbac157 36542452 PMC10016409

[B36] Mémoli F. (2011). Gromov–wasserstein distances and the metric approach to object matching. *Foundations Comput. Math.* 11 417–487. 10.1007/s10208-011-9093-5

[B37] Michalke L. Dreyer A. M. Borst J. P. Rieger J. W. (2023). Inter-individual single-trial classification of MEG data using M-CCA. *NeuroImage* 273:120079. 10.1016/j.neuroimage.2023.120079 37023989

[B38] Mikolov T. Sutskever I. Chen K. Corrado G. Dean J. (2013). Distributed representations of words and phrases and their compositionality. *arxiv* [Preprint] 10.48550/arXiv.1310.4546

[B39] Miyawaki Y. Uchida H. Yamashita O. Sato M. Morito Y. Tanabe H. C. (2008). Visual image reconstruction from human brain activity using a combination of multiscale local image decoders. *Neuron* 60 915–929. 10.1016/j.neuron.2008.11.004 19081384

[B40] Nielsen F. (2016). *Hierarchical Clustering. In Introduction to HPC with MPI for Data Science.* Berlin: Springer, 195–211.

[B41] Nishida S. Matsumoto Y. Yoshikawa N. Son S. Murakami A. Hayashi R. (2020). Reduced intra- and inter-individual diversity of semantic representations in the brains of schizophrenia patients. *BioRxiv* [Preprint] 10.1101/2020.06.03.132928

[B42] Nishimoto S. Vu A. T. Naselaris T. Benjamini Y. Yu B. Gallant J. L. (2011). Reconstructing visual experiences from brain activity evoked by natural movies. *Curr. Biol.* 21 1641–1646. 10.1016/j.cub.2011.08.031 21945275 PMC3326357

[B43] Okazawa G. Tajima S. Komatsu H. (2015). Image statistics underlying natural texture selectivity of neurons in macaque V4. *Proc. Natl. Acad. Sci.* 112 E351–E360. 10.1073/pnas.1415146112 25535362 PMC4313822

[B44] Oord A. Van Den Li Y. Vinyals O. (2018). Representation learning with contrastive predictive coding. *arxiv* [Preprint] 10.48550/arXiv.1807.03748

[B45] Ozcelik F. VanRullen R. (2023). Natural scene reconstruction from fMRI signals using generative latent diffusion. *Sci. Rep.* 13:15666. 10.1038/s41598-023-42891-8 37731047 PMC10511448

[B46] Pais-Vieira M. Lebedev M. Kunicki C. Wang J. Nicolelis M. A. L. (2013). A Brain-to-brain interface for real-time sharing of sensorimotor information. *Sci. Rep.* 3:1319. 10.1038/srep01319 23448946 PMC3584574

[B47] Penfield W. Boldrey E. (1937). Somatic motor and sensory representation in the cerebral cortex of man as studied by electrical stimulation. *Brain* 60 389–443. 10.1093/brain/60.4.389

[B48] Quadrianto N. Smola A. J. Song L. Tuytelaars T. (2010). Kernelized Sorting. *IEEE Trans. Pattern Anal. Mach. Intell.* 32 1809–1821. 20724758 10.1109/TPAMI.2009.184

[B49] Romani G. L. Williamson S. J. Kaufman L. (1982). Tonotopic organization of the human auditory cortex. *Science* 216 1339–1340. 10.1126/science.7079770 7079770

[B50] Rombach R. Blattmann A. Lorenz D. Esser P. Ommer B. (2022). “High-resolution image synthesis with latent diffusion models,” in *Proceedings of the 2022 IEEE/CVF Conference on Computer Vision and Pattern Recognition (CVPR), 2022-June*, (Piscataway, NJ: IEEE), 10674–10685. 10.1088/1361-6560/ad229e

[B51] Saharia C. Ho J. Chan W. Salimans T. Fleet D. J. Norouzi M. (2023). Image super-resolution via iterative refinement. *IEEE Trans. Pattern Anal. Mach. Intell.* 45 4713–4726. 10.1109/TPAMI.2022.3204461 36094974

[B52] Shen G. Dwivedi K. Majima K. Horikawa T. Kamitani Y. (2019a). End-to-end deep image reconstruction from human brain activity. *Front. Comput. Neurosci.* 13:12. 10.3389/fncom.2019.00021 31031613 PMC6474395

[B53] Shen G. Horikawa T. Majima K. Kamitani Y. (2019b). Deep image reconstruction from human brain activity. *PLoS Comput. Biol.* 15:e1006633. 10.1371/journal.pcbi.1006633 30640910 PMC6347330

[B54] Sohl-Dickstein J. Weiss E. A. Maheswaranathan N. Ganguli S. (2015). Deep unsupervised learning using nonequilibrium thermodynamics. *arxiv* [Preprint] 10.48550/arXiv.1503.03585

[B55] Takagi Y. Nishimoto S. (2023). “High-resolution image reconstruction with latent diffusion models from human brain activity,” in *Proceedings of the 2023 IEEE/CVF Conference on Computer Vision and Pattern Recognition (CVPR)*, (Piscataway, NJ: IEEE), 14453–14463.

[B56] Takahashi S. Sasaki M. Takeda K. Oizumi M. (2024). “Self-supervised learning facilitates neural representation structures that can be unsupervisedly aligned to human behaviors,” in *Proceedings of the ICLR 2024 Workshop on Representational Alignment*, (ICLR), 1–12.

[B57] Takeda K. Abe K. Kitazono J. Oizumi M. (2024). “Unsupervised alignment reveals structural commonalities and differences in neural representations of natural scenes across individuals and brain areas,” in *Proceedings of the ICLR 2024 Workshop on Representational Alignment*, (ICLR), 1–15. 10.1016/j.isci.2025.112427 PMC12059663 40343275

[B58] Wandell B. A. Brewer A. A. Dougherty R. F. (2005). Visual field map clusters in human cortex. *Philos. Trans. R. Soc. B Biol. Sci.* 360 693–707. 10.1098/rstb.2005.1628 15937008 PMC1569486

[B59] Wang T. Isola P. (2020). “Understanding contrastive representation learning through alignment and uniformity on the hypersphere,” in *Proceedings of the 37th International Conference on Machine Learning, ICML 2020, PartF16814*, (ICLM), 9871–9881.

[B60] Wen H. Shi J. Zhang Y. Lu K.-H. Cao J. Liu Z. (2018). Neural encoding and decoding with deep learning for dynamic natural vision. *Cereb. Cortex* 28 4136–4160. 10.1093/cercor/bhx268 29059288 PMC6215471

[B61] Wu Z. Xiong Y. Yu S. X. Lin D. (2018). “Unsupervised feature learning via non-parametric instance discrimination,” in *Proceedings of the IEEE Computer Society Conference on Computer Vision and Pattern Recognition*, (Piscataway, NJ: IEEE), 3733–3742.

[B62] Yamada M. Sugiyama M. (2011). Cross-domain object matching with model selection. *Proc. Mach. Learn. Res*. 15, 807–815.

[B63] Yoo S.-S. Bystritsky A. Lee J.-H. Zhang Y. Fischer K. Min B.-K. (2011). Focused ultrasound modulates region-specific brain activity. *NeuroImage* 56 1267–1275. 10.1016/j.neuroimage.2011.02.058 21354315 PMC3342684

[B64] Zhu J.-Y. Park T. Isola P. Efros A. A. (2017). “Unpaired image-to-image translation using cycle-consistent adversarial networks,” in *Proceedings of the 2017 IEEE International Conference on Computer Vision (ICCV), 2017-Octob*, (Piscataway, NJ: IEEE), 2242–2251.

