# Supplementary material for: Unsupervised method for representation transfer from one brain to another
Source: Front Neuroinform. 2024 Nov 28;18:1470845. doi: 10.3389/fninf.2024.1470845 (PMC11634869; doi:10.3389/fninf.2024.1470845)
Supplement: Supplementary file 1 [file Data_Sheet_1.docx]

Supplementary Material

# Grid search of hyperparameters in Section 2.2.1

In the instance learning step, there are hyperparameters τ, which adjusts the repulsion between the positive and negative samples, and *kσ*, which controls the noise gain during data augmentation. We used the same loss function in instance learning as the official implementation by Wu et al. (https://github.com/zhirongw/lemniscate.pytorch), and the repulsion parameter τ was the same as that used in their paper (Wu et al., 2018). Supplementary Figure 1 illustrates the change in the distribution of cosine similarities between the latent variables of the test data pairs based on the choice of hyperparameter values in the instance learning step. As depicted in Supplementary Figure 1A, the histogram of the pairwise cosine similarities tended to distribute symmetrically around zero, with a skewness close to zero (0.0767) when both the repulsion parameter τ and the noise gain parameter *kσ* were set to small values, specifically 0.2 and 0.5, respectively. The result of this distribution suggests that the data samples were uniformly embedded on the hypersphere, which collapsed the relationships based on the similarities or dissimilarities of the original data. However, when a larger τ value (i.e., 4) was used during training, the distribution became more asymmetrical, as evidenced by a positive skewness value (0.4592, Supplementary Figure 1B). Furthermore, the mean value of the distribution shifted toward negative values, while displaying a long-tailed distribution toward positive values. These distribution properties suggested that similar test samples were embedded closer together, while dissimilar test samples were embedded further apart on the surface of the hypersphere compared with the results obtained using smaller τ values in the instance learning step. A similar trend was observed (skewness = 0.5133) when using a larger *kσ* value (i.e., 2) instead of a larger τ value (Supplementary Figure 1C). Therefore, hyperparameter selection is crucial for embedding data on the hypersphere while preserving the relationships based on the similarities or dissimilarities of the original data, which is essential for the subsequent representation transfer procedure.


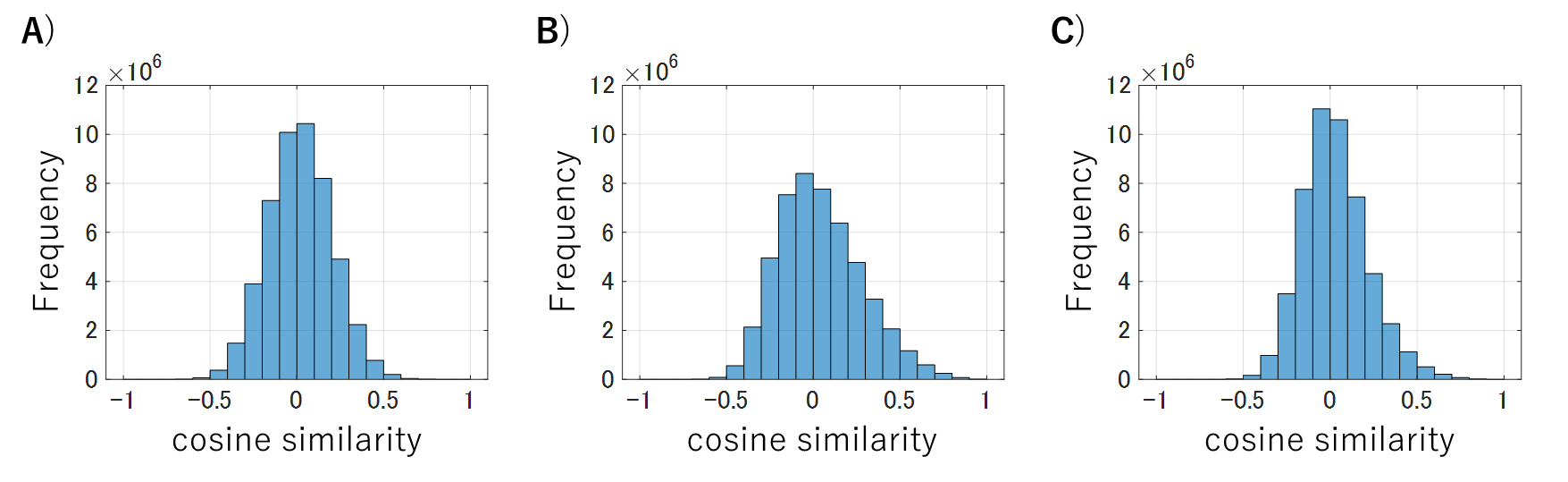


**Supplementary Figure 1.** Histograms depicting the cosine similarities between the latent variables of all test data pairs under different hyperparameter values used for embedding data on the hypersphere according to the instance learning rule. **(A)** Histogram of the results when using (τ, *kσ*) = (0.2, 0.5), resulting in a skewness of 0.0767. **(B)** Histogram of the results when using (τ, *kσ*) = (4, 0.5), resulting in a skewness of 0.4592. **(C)** Histogram of the results when using (τ, *kσ*)= (0.2, 2), resulting in a skewness of 0.5133.

To determine the hyperparameters that optimize data embedding for the subsequent representation transfer procedure, we performed a grid search for both the repulsion parameter τ and the noise gain parameter *kσ*. The objective was to achieve a high skewness value in the pairwise cosine similarity distribution between the latent variables and also ensure that the embedded data become linearly independent within the range of machine precision when subjected to singular value decomposition (please refer to the main text for the rationale behind this criteria). For the grid search, we set τ ∈ {0.1, 0.2, 0.3, 0.5, 0.8, 1} and *kσ* ∈{0, 0.25, 0.5, 0.75, 1, 1.5, 1.75, 2}. Supplementary Figure 2 depicts the results of the grid search for the datasets with different dimensions when embedding them into a hypersphere in a 32-dimensional space. The top row of Supplementary Figure 2 shows the skewness value of the resulting distribution in the form of contour maps, and the bottom row shows the linear independence of the latent variables at each search point. The skewness of the resulting distribution tended to increase as τ and *kσ* increased. However, setting τ and *kσ* too high led to linear dependence among the latent variables, which indicated that the data were not distributed across the full dimensions of the hypersphere and were excessively aggregated. Although we have only presented the results of one ANN in Supplementary Figure 2, we observed similar grid search results for the dependency of the two hyperparameters when using datasets obtained using several different ANN models. Therefore, we determined the optimal τ and *kσ* values after the grid search using one dataset for each tested dimension and applied these values to the other datasets for subsequent training.


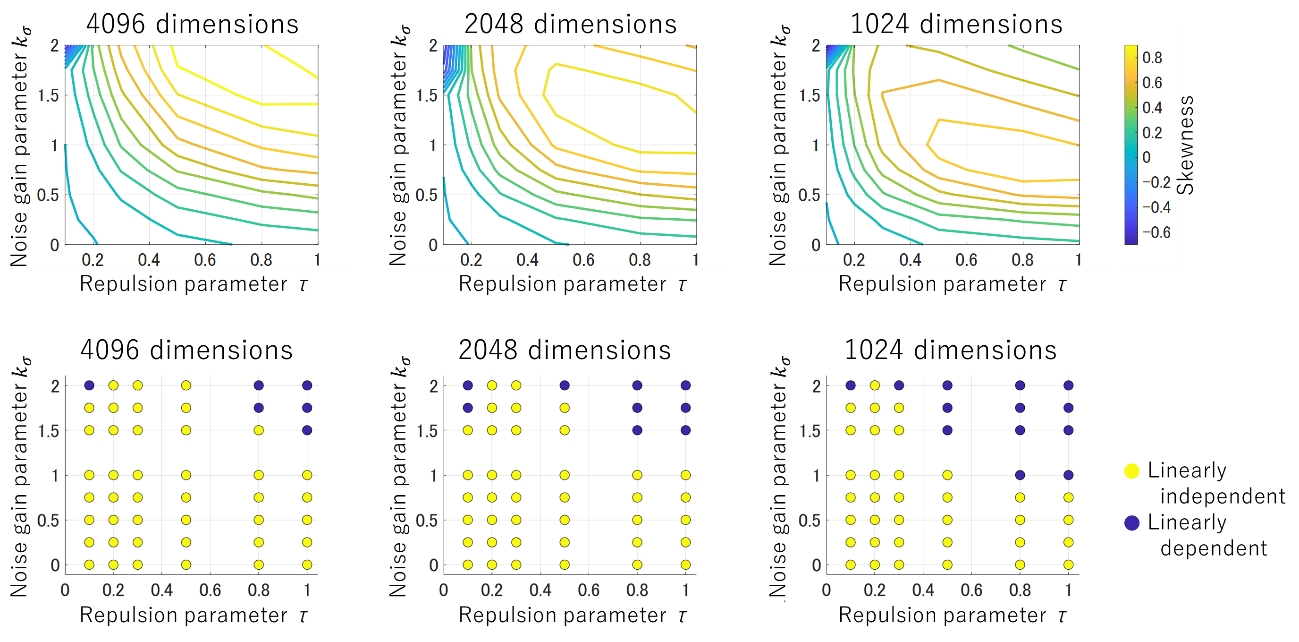


**Supplementary Figure 2.** Results of the grid search for both the repulsion parameter τ and the noise gain parameter *kσ*. Left column: Results obtained when embedding a dataset of 4096 dimensions from an intermediate layer of one ANN model into a hypersphere in a 32-dimensional space. Center column: Results obtained when embedding a dataset of 2048 dimensions. Right column: Results obtained when embedding a dataset of 1024 dimensions. Top row: Contour plots showing the skewness value of the resulting distributions for each data dimension. Bottom row: Visualization of the linear independence of the latent variables within the range of machine precision when subjected to singular value decomposition for each search point. Yellow indicates that the resulting data distribution was linearly independent, and blue indicates linear dependence.

# Inter-ANN representation transfer

## Relationship between the HSIC values in kernelized sorting and alignment scores based on the sorting results

We observed that the alignment scores between some pairs of ANNs were lower than those between other pairs that reached close to the upper bound of alignment performance (i.e., the alignment score that could be achieved with the use of corresponding label information). To understand this discrepancy, we scrutinized the kernelized sorting process and plotted how the HSIC value (which is used by kernelized sorting to determine the match) relates to the alignment score determined by the obtained matching results. Supplementary Figure 3 shows the scatter plots of the alignment scores and HSIC values for each sorting. Each panel corresponds to the different pairs of ANN models and the varying numbers of dimensions of the dataset (Supplementary Figure 3A, B, and C correspond to the results of the 4096-, 2048-, and 1024-dimension datasets, respectively). The top left panel of Supplementary Figure 3A corresponds to the results shown in Figure 3C as representative data. The value of r at the top of each panel represents the correlation coefficient between the alignment score and the HSIC values. All results showed positive correlation coefficients higher than 0.30, which indicated that using the sorting results corresponding to higher HSIC values led to a greater likelihood of obtaining higher alignment scores in the representation transfer. However, the sorting with the highest HSIC values did not necessarily result in the highest alignment score.


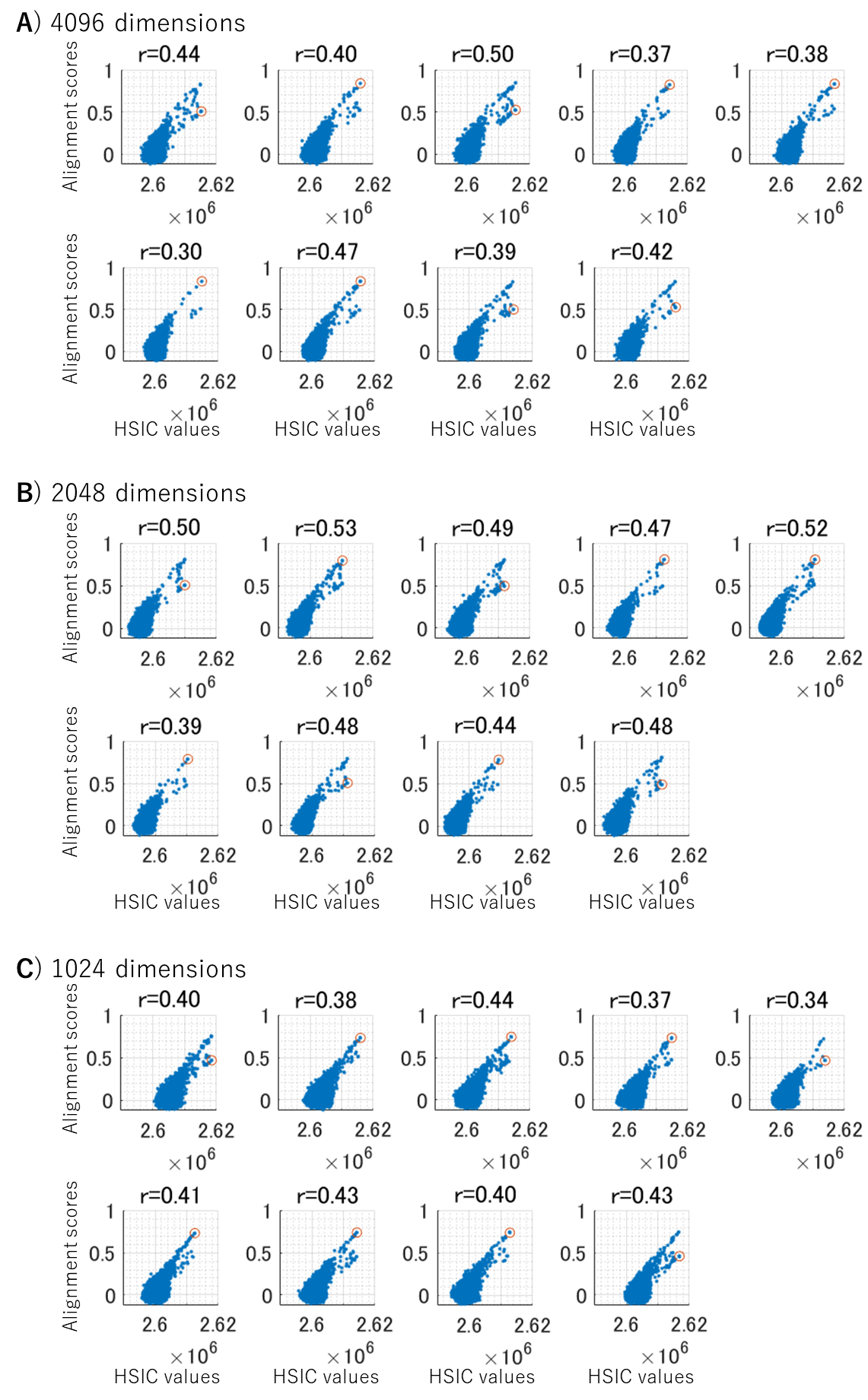


**Supplementary Figure 3.** Scatter plots depicting the alignment scores and HSIC values for the different pairs of datasets. Each panel corresponds to the different pairs of ANN models and varying numbers of dimensions of the dataset. **(A)** Results from the 4096-dimension dataset. **(B)** Results from the 2048-dimension dataset. **(C)** Results from the 1024-dimension dataset. The horizontal axis of each panel represents the HSIC value, and the vertical axis represents the alignment scores. Each blue point in the panel corresponds to one sorting result. The red circle indicates the sorting result with the highest HSIC value.

## The distribution of cosine similarities between the latent variables of one ANN model and those transferred from another ANN

We calculated the cosine similarities between the latent variables of the test data for one ANN model and the corresponding data transferred from another ANN using our proposed method to explore how the data were aligned between the two datasets in several cases that resulted in dissociated alignment scores. Supplementary Figure 4 shows a histogram of the cosine similarities between 5000 test data pairs for different pairs of ANNs with two different dimensions. The top row illustrates the results from pairs of datasets that ended with high alignment scores, and the bottom row illustrates the results from pairs of datasets that ended with low alignment scores.

The panels in the top row with high alignment scores demonstrate that the cosine similarities of the corresponding test data were distributed around 1 with low variance. This indicated that the data samples from another ANN were transferred close to the corresponding samples in the latent space for the target ANN. In contrast, the panels in the bottom row with low alignment scores illustrate that the cosine similarities of the corresponding test data were distributed with a higher variance; moreover, a large number of samples exhibited similarities that were lower than 0.5.

The latter results suggest that our proposed method aligned the two datasets by matching merely the overall distribution of the two datasets when kernelized sorting was unable to extract sufficiently correct matches needed for accurate alignment. Furthermore, samples distributed away from the circular mean direction of the spherical distribution showed larger alignment dissociations.


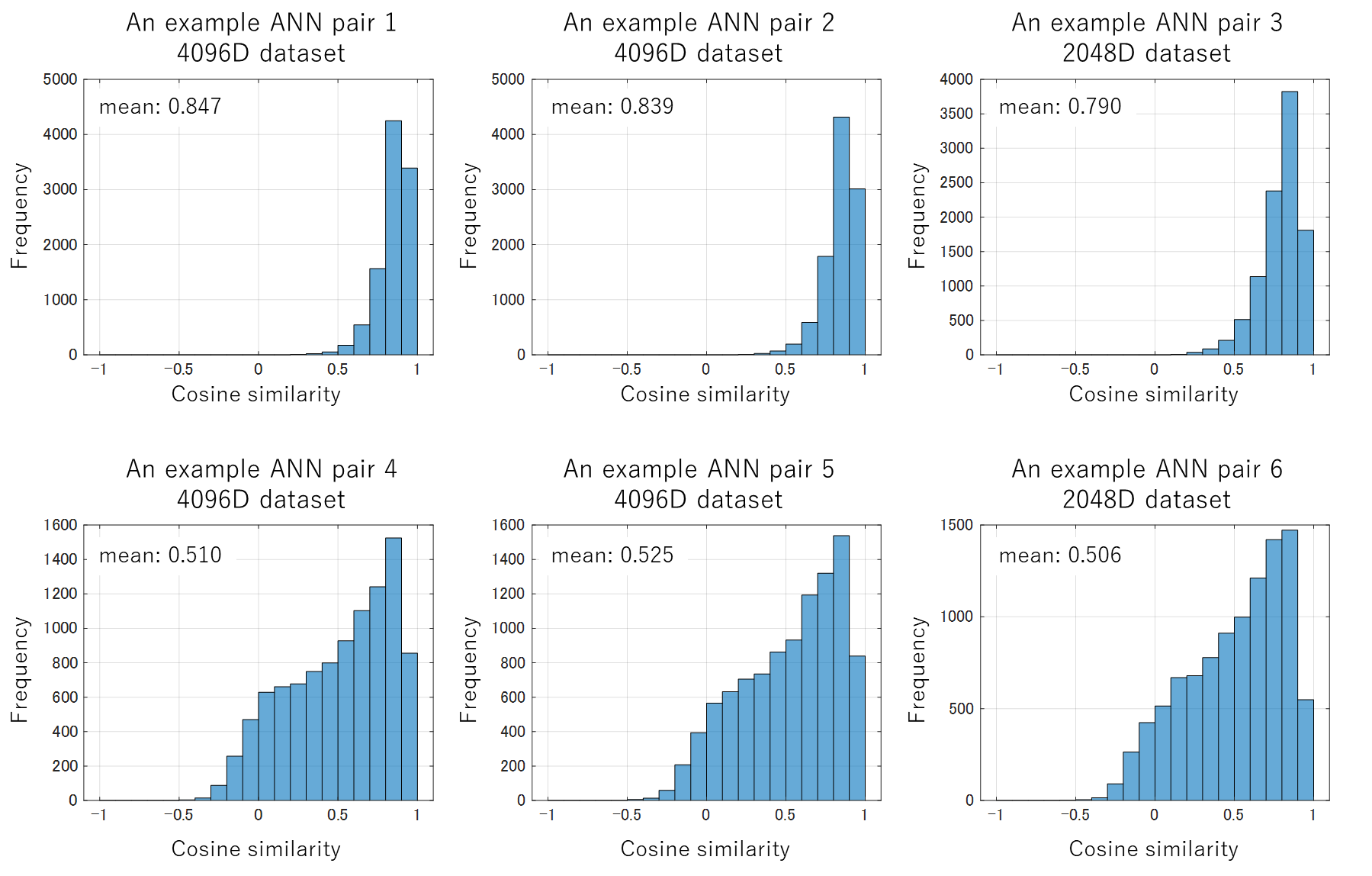


**Supplementary Figure 4.** The distribution of the cosine similarities between the latent variables of the test data for one ANN model and corresponding test data transferred from another ANN. Top row: Histogram plots of the results of the example pairs of datasets that produced high alignment scores. Bottom row: Histogram plots of the results of the example pairs of datasets that produced low alignment scores. Different panels represent results using different ANN pairs or datasets with different dimensions.

## Relationship between the residual of the orthogonal matrix estimation and the alignment score

It is conceivable that the alignment scores were low in some pairs of ANNs because of an incorrect calculation of the orthogonal matrix based on the matched representative points obtained following the kernelized sorting step. To examine this possibility, we calculated the residual of the orthogonal matrix estimation as $\left\| R-YX^{T} \right\|_{F}$ (Equation S1). If the transferred representative points after orthogonal transformation (i.e., *RX*) and the target representative points *Y* are aligned perfectly, then it would lead to the equation $RX=Y$ (Equation S2). Therefore, we defined the mean square errors between the estimated orthogonal matrix and the inner product of *Y* and *X^T^*, which was derived by multiplying both sides of Equation S2 by *X^T^* (the inverse of *X*), to obtain the difference.

Supplementary Figure 5 illustrates the relationship between the residual of the orthogonal matrix estimation and the alignment score obtained using the estimated orthogonal matrix. The results indicated that some pairs of ANNs resulted in low alignment scores even though the residual of the orthogonal matrix estimation was low, and vice versa (i.e., high alignment scores achieved under a high residual of the orthogonal matrix estimation). Therefore, the estimation method of the orthogonal matrix based on the matched representative points is not critical to producing a low alignment score; rather, the match between representative points per se, derived from kernelized sorting because of the fall in the local optima, is critical.


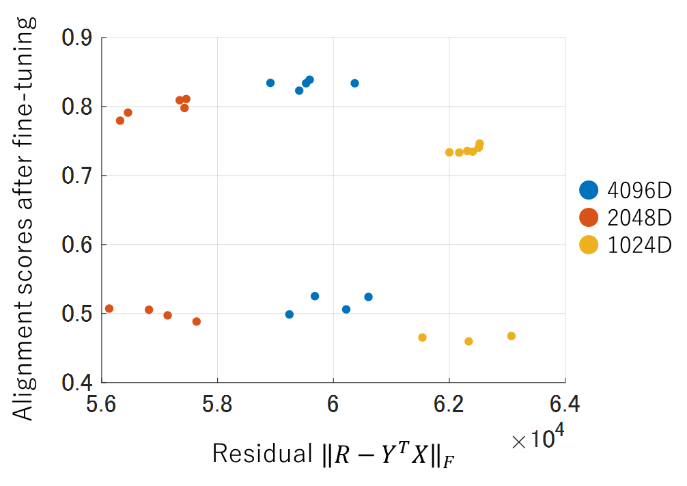


**Supplementary Figure 5.** The relationship between the residual of the orthogonal matrix estimation and the alignment score. The horizontal axis indicates the residual of the orthogonal matrix estimation, and the vertical axis indicates the alignment score obtained after representation transfer using the estimated orthogonal matrix. Each point in the figure represents the result of each data pair from different ANNs. The colors represent different dimension sizes of the data.

## Overlap of image category clusters across representation transfer

In Supplementary Figure 6, the latent variables from a pair of ANNs that exhibited a low alignment score were visualized in the same two-dimensional plane using the Uniform Manifold Approximation and Projection (UMAP) method. Each panel depicts the correspondence of test samples from one image category with test samples transferred to the nearest location, connected by colored lines. In a perfect alignment scenario, test samples from one image category connect with those of the same category from the other ANN. Our results suggest that representation transfer achieved partial overlap of the clustering structure of image categories, despite an overall low alignment score for this ANN pair.


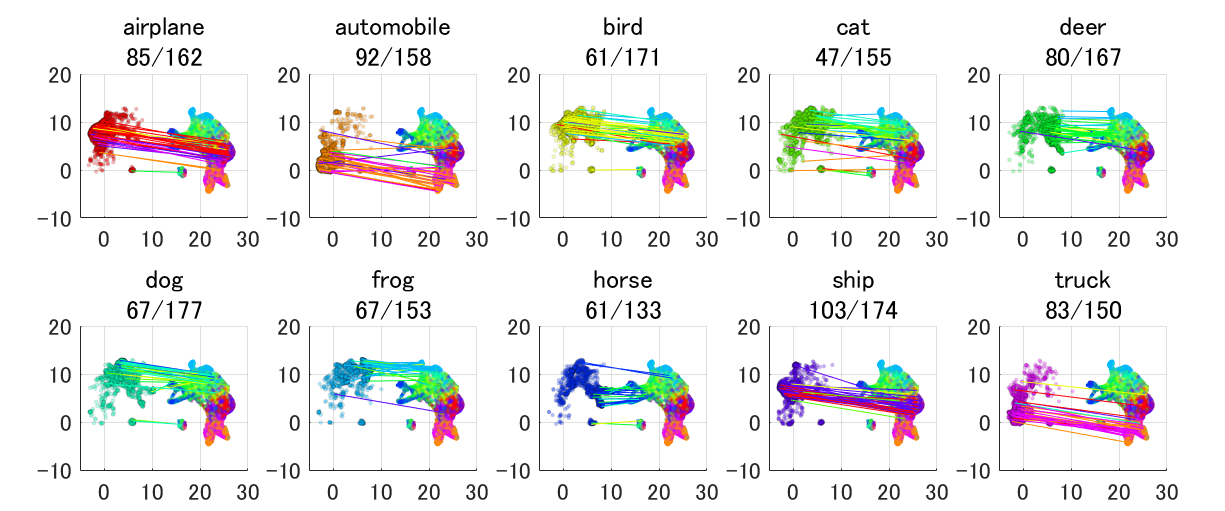


**Supplementary Figure 6.** Visualization of the latent variables of two ANNs. Each panel represents the same Uniform Manifold Approximation and Projection (UMAP) plot derived from the data of a specific pair of ANNs. Each panel shows the correspondence between the latent variables of the two ANNs for only one image category, shown as connected lines. Each point denotes a test sample, with different colors representing different image categories (shown at the top of each panel). The number displayed at the top of each panel indicates the ratio of samples matched to the same image category.

# Method for reconstructing input images from latent variables

Supplementary Table 1 provides the details of the network architecture of the decoder, which reconstructed the input images from the latent variables embedded by the encoder. The data flows from the coarse-scale layer, shown in the top row, to the fine-scale layer at the bottom. We trained the decoder using the encoder described in the main text as the interface from the input images to the latent variables. The learnable parameters of the encoder were fixed during the training of the decoder. Initially, we trained the coarse-scale layer and the fine-scale layer separately using mean squared error loss: the loss between the input of the encoder and the output of the decoder was used to train the coarse-scale layer, whereas pixel loss (i.e., the loss between the input images and the reconstructed images) was used to train the fine-scale layer. Thereafter, we fine-tuned both layers together using pixel loss.

Supplementary Table 1. The network architecture of the decoder

|  | Modules | Dimensions of input and output variables of each layer | Parameters |
| --- | --- | --- | --- |
| Coarse-scale layer | Fully connected layer with Batch norm. + ReLU | 32 to 362 | Bias=False |
|  | Fully connected layer with Batch norm. + ReLU | 362 to 4096 | Bias=False |
| Fine-scale layer | Fully connected layer  with reshape | 4096 to 16×16×32 |  |
|  | Deconvolution layer with Batch norm. + ReLU | 16×16×32 to 32×32×16 | Kernel size 4×4, Stride 2, Padding 1 |
|  | Deconvolution layer with Batch norm. + ReLU | 32×32×16 to 32×32×16 | Kernel size 3×3, Stride 1, Padding 1 |
|  | Deconvolution layer with Batch norm. + tanh | 32×32×16 to 32×32×3 | Kernel size 3×3, Stride 1, Padding 1 |

The images generated by the trained decoder appeared blurry (refer to the bottom rows of Supplementary Figure 6). To improve the quality of reconstructed images so that they appear as natural photographic images, we applied additional image refinement processing to the decoder’s output. We used an unofficial PyTorch implementation of a method called image super-resolution via repeated refinement (SR3) (Saharia et al., 2023). The parameter setting for the diffusion model used in this refinement method was sourced from an open repository (<https://github.com/Janspiry/Image-Super-Resolution-via-Iterative-Refinement> ) in a parameter file named sr_sr3_16_128.json. We adjusted the parameter “l_resolution” from 16 to 32, “r_resolution” from 128 to 32, and “batch_size” from 4 to 1024 and applied the refinement processing to the decoder’s output images.

# Quantitative evaluation of reconstructed images

We quantitatively evaluated the resemblance of the images generated by the decoder to the original images, followed by refinement processing. We conducted several evaluations of the images reconstructed from the latent variables output by the encoder of one ANN (i.e., without representation transfer) and latent variables transferred from another ANN (i.e., with inter-ANN representation transfer). Owing to the time-consuming nature of decoder training and image refinement, we tested images using only a single pair of ANNs.

The L2 norm difference between the original and reconstructed images without representation transfer yielded mean and standard deviation values of 552.4 and 150.4, respectively, across 5000 test images. With inter-ANN representation transfer, the mean and standard deviation of the L2 norm difference were 536.0 and 165.1, respectively.

We then used a pre-trained ResNet20 model to classify the 10 image categories of the CIFAR-10 dataset and evaluated its accuracy in classifying the reconstructed images according to the original image categories. The model achieved a 10-category classification accuracy of 92.59% for the test images, demonstrating its suitability for this task. The classification accuracy for the reconstructed images without representation transfer and with inter-ANN representation transfer was 57.72% and 45.88%, respectively. These results indicated significant degradation in image quality due to the limitations of the decoder, although the distortion caused by representation transfer was less pronounced. The agreement in image categories classified by the ResNet20 model between the images without representation transfer and the corresponding images with inter-ANN representation transfer was 47.47%. This indicated that nearly half of the generated images were classified into the same image categories even after representation transfer, which was substantially above chance (10%).

Finally, we assessed whether the image refinement procedure using the diffusion model improved the quality of reconstructed images by comparing the Frechet Inception Distance (FID) before and after refinement. Lower FID values indicate better image quality. FID values decreased after refinement in both cases: from 160.509 to 28.4187 without representation transfer and from 150.779 to 34.8891 with inter-ANN representation transfer. These results confirmed the effectiveness of the implemented image refinement procedure using the diffusion model.


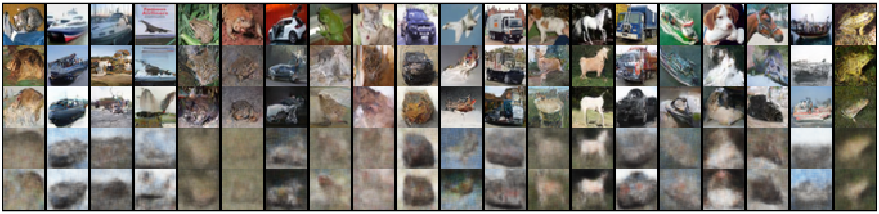


**Supplementary Figure 7.** Examples of the reconstructed images for 20 different test images. The top row shows the test images. The second and third rows show the reconstructed images after refinement without representation transfer and with inter-ANN transfer, respectively. The fourth and fifth rows display the reconstructed images before refinement without representation transfer and with inter-ANN transfer, respectively.

# Relationship between inter-participant representation similarity and alignment score in the brain word representation data

The results shown in Figure 5A revealed variations in the performance of the proposed representation transfer across different participant pairs when using brain word representation data. To examine the factors contributing to this variability in transfer performance, we assessed the representational similarity between participants using their corresponding labels. We found that participant pairs that had low alignment scores also exhibited low representational similarity, as determined by correlation coefficients between RDMs. When using RDMs derived from pairwise Euclidean distances of brain word representations, the correlation coefficients for all participant pairs between alignment scores and similarity between RDMs was 0.710. Similarly, when using RDMs derived from pairwise cosine distances of latent variables, the correlation coefficient was 0.774.


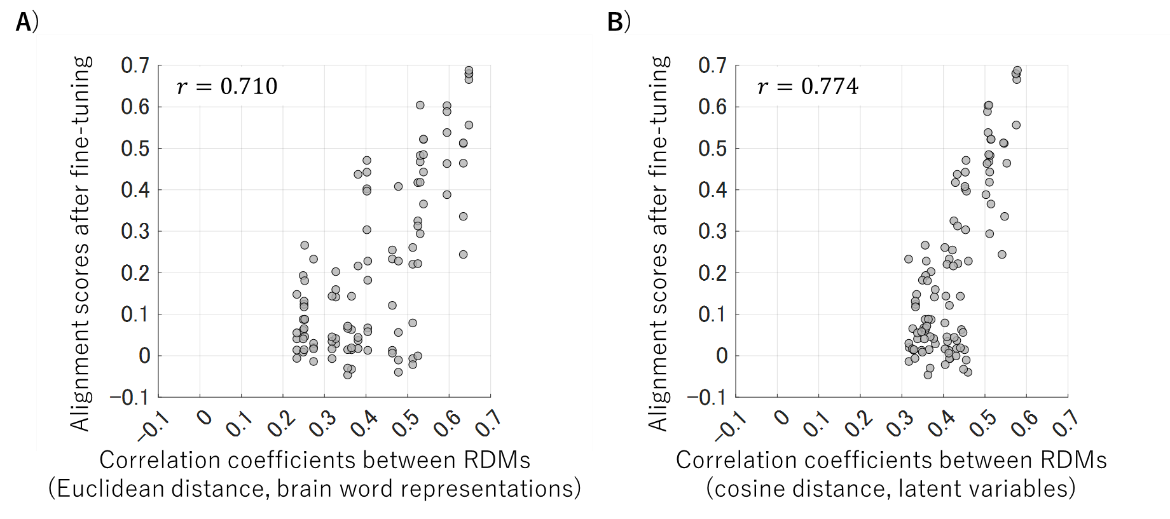


**Supplementary Figure 8.** Relationship between inter-participant representation similarity and alignment score in brain word representation data. Inter-participant representation similarity was assessed using two methods (as indicated in (A) and (B)). The correlation coefficients between these two factors were 0.710 and 0.774 for (A) and (B). Each point represents the result for each participant pair. The alignment scores were calculated after the fine-tuning step of our proposed method. **(A)** The horizontal axis represents the correlation coefficient between the RDMs defined by the pairwise Euclidian distances of brain word representations from two participants, and the vertical axis represents the alignment score. **(B)** The horizontal axis represents the correlation coefficient between the RDMs defined by the pairwise cosine distances of the embedded latent variables of two participants’ brain word representations, and the vertical axis represents the alignment score.
